# Metatranscriptomic and Thermodynamic Insights into Medium-Chain Fatty Acid Production Using an Anaerobic Microbiome

**DOI:** 10.1128/mSystems.00221-18

**Published:** 2018-11-20

**Authors:** Matthew J. Scarborough, Christopher E. Lawson, Joshua J. Hamilton, Timothy J. Donohue, Daniel R. Noguera

**Affiliations:** a The Great Lakes Bioenergy Research Center, University of Wisconsin—Madison, Madison, Wisconsin, USA; b Department of Civil and Environmental Engineering, University of Wisconsin—Madison, Madison, Wisconsin, USA; c Department of Bacteriology, University of Wisconsin—Madison, Madison, Wisconsin, USA; d Department of Biochemistry, University of Wisconsin—Madison, Madison, Wisconsin, USA; Ghent University

**Keywords:** medium-chain fatty acids, hexanoic acid, octanoic acid, carboxylate platform, anaerobic digestion, biorefining, metagenomics, metatranscriptomics

## Abstract

Biomanufacturing from renewable feedstocks can offset fossil fuel-based chemical production. One potential biomanufacturing strategy is production of medium-chain fatty acids (MCFA) from organic feedstocks using either pure cultures or microbiomes. While the set of microbes in a microbiome can often metabolize organic materials of greater diversity than a single species can and while the role of specific species may be known, knowledge of the carbon and energy flow within and between organisms in MCFA-producing microbiomes is only now starting to emerge. Here, we integrated metagenomic, metatranscriptomic, and thermodynamic analyses to predict and characterize the metabolic network of an anaerobic microbiome producing MCFA from organic matter derived from lignocellulosic ethanol fermentation conversion residue. A total of 37 high-quality (>80% complete, <10% contamination) metagenome-assembled genomes (MAGs) were recovered from the microbiome, and metabolic reconstruction of the 10 most abundant MAGs was performed. Metabolic reconstruction combined with metatranscriptomic analysis predicted that organisms affiliated with *Lactobacillus* and *Coriobacteriaceae* would degrade carbohydrates and ferment sugars to lactate and acetate. *Lachnospiraceae*- and *Eubacteriaceae*-affiliated organisms were predicted to transform these fermentation products to MCFA. Thermodynamic analyses identified conditions under which H_2_ is expected to be either produced or consumed, suggesting a potential role of H_2_ partial pressure in MCFA production. From an integrated systems analysis perspective, we propose that MCFA production could be improved if microbiomes were engineered to use homofermentative instead of heterofermentative *Lactobacillus* and if MCFA-producing organisms were engineered to preferentially use a thioesterase instead of a coenzyme A (CoA) transferase as the terminal enzyme in reverse β-oxidation.

**IMPORTANCE** Mixed communities of microbes play important roles in health, the environment, agriculture, and biotechnology. While tapping the combined activities of organisms within microbiomes may allow the utilization of a wider range of substrates in preference to the use of pure cultures for biomanufacturing, harnessing the metabolism of these mixed cultures remains a major challenge. Here, we predicted metabolic functions of bacteria in a microbiome that produces medium-chain fatty acids from a renewable feedstock. Our findings lay the foundation for efforts to begin addressing how to engineer and control microbiomes for improved biomanufacturing, how to build synthetic mixtures of microbes that produce valuable chemicals from renewable resources, and how to better understand the microbial communities that contribute to health, agriculture, and the environment.

**Author Video**: An author video summary of this article is available.

## Abstract (precis)

Mixed communities of microbes play important roles in health, the environment, agriculture, and biotechnology. While tapping the combined activities of organisms within microbiomes may allow the utilization of a wider range of substrates in preference to the use of pure cultures for biomanufacturing, harnessing the metabolism of these mixed cultures remains a major challenge. Here, we predicted metabolic functions of bacteria in a microbiome that produces medium-chain fatty acids from a renewable feedstock. Our findings lay the foundation for efforts to begin addressing how to engineer and control microbiomes for improved biomanufacturing, how to build synthetic mixtures of microbes that produce valuable chemicals from renewable resources, and how to better understand the microbial communities that contribute to health, agriculture, and the environment.

## INTRODUCTION

Biological production of chemicals from renewable resources is an important step to reduce societal dependence on fossil fuels. One approach that shows potential for the biological production of chemicals from renewable resources, represented by the carboxylate platform (1, 2), uses anaerobic microbial communities to biotransform complex substrates into carboxylic acids, including medium-chain fatty acids (MCFA). MCFA such as hexanoate (a six-carbon [C6] monocarboxylate) and octanoate (an eight-carbon [C8] monocarboxylate) are used in large quantities for the production of pharmaceuticals, antimicrobials, and industrial materials and can be processed to form chemicals currently derived from fossil fuels (3, 4).

Previous applications of the carboxylate platform have focused on converting organics from undistilled corn beer (5, 6), food (7, 8), winery residue (9), thin stillage from corn ethanol production (10), and lignocellulose-derived materials (11–13) to MCFA, and, as we have previously shown for lignocellulosic biofuel production (4), one can anticipate the economic benefits of converting organic residues from these industries into MCFA.

MCFA-producing bioreactors contain diverse microbial communities (4, 5, 12). While the roles of some community members in these microbiomes can be inferred from studies performed with pure cultures and from phylogenetic relationships (10, 12, 14, 15), detailed knowledge of specific metabolic activities in many members of these microbiomes is only starting to emerge (16). In general, some community members participate in hydrolysis and fermentation of available organic substrates, while others are involved in the conversion of intermediates to MCFA via reverse β-oxidation, a process also known as chain elongation (1). In reverse β-oxidation, an acyl-coenzyme A (acyl-CoA) unit is combined with acetyl-CoA, with each cycle elongating the resulting carboxylic acid by two carbons (1). Energy conservation in organisms using reverse β-oxidation as the main metabolic process for growth relies on ATP generation with reduced ferredoxin, which is generated through both pyruvate ferredoxin oxidoreductase and an electron-bifurcating acyl-CoA dehydrogenase (17). A proton translocating ferredoxin-NAD reductase is used to reduce NAD with ferredoxin and create an ion motive force which is used to generate ATP (17). The even-chain butyric (C4), hexanoic (C6), and octanoic (C8) acids are all potential products of reverse β-oxidation under conditions in which the process is initiated with acetyl-CoA. The odd-chain valeric (C5) and heptanoic (C7) acids are products of reverse β-oxidation under conditions in which the chain elongation process starts with propionyl-CoA. While there have been demonstrations of this wide range of possible products from chain elongation (5, 18) and while MCFA bioreactors typically produce more than one product (4, 12, 14, 15, 19, 20), a strategy to control the final product length has not yet emerged. We are interested in obtaining the knowledge needed for the rational development and implementation of strategies to improve MCFA yields and control product formation in MCFA-producing microbiomes.

We reported earlier on a bioreactor that produced a mixture of acetate, C4, C6, and C8 from lignocellulosic stillage (4). On the basis of 16S rRNA tag sequencing, we found that five major genera, including three *Firmicutes* genera (*Lactobacillus*, *Roseburia*, and *Pseudoramibacter*) and two *Actinobacteria* genera (*Atopobium* and *Olsenella*), represented more than 95% of the community (4). On the basis of the phylogenetic association of these organisms, the *Lactobacillus* and the *Actinobacteria* were hypothesized to produce lactic acid, while *Roseburia* and *Pseudoramibacter* were hypothesized to produce the even-chain C4, C6, and C8 acids (4). Furthermore, lactic acid was proposed to be the key fermentation product that initiated chain elongation in the microbiome (4). However, since knowledge of phylogenetic associations is not enough to enable detailed understanding of the metabolism of these organisms, the earlier study did not generate sufficient knowledge to help understand how to control a MCFA-producing microbiome.

Here we report on further studies of the MCFA-producing microbiome reported earlier (4), where we utilized a combination of metagenomic, metatranscriptomic, and thermodynamic analyses to reconstruct the combined metabolic activity of the microbial community. We analyzed the gene expression patterns of the 10 most abundant community members during steady-state reactor operation. Our results identified several community members that expressed genes predicted to be involved in complex carbohydrate degradation and in the subsequent fermentation of degradation products to lactate and acetate. Genes encoding enzymes for reverse β-oxidation were expressed by two abundant organisms affiliated with the class *Clostridia*. On the basis of a thermodynamic analysis of the proposed MCFA-producing pathways, we suggest that individual clostridial organisms use different substrates for MCFA production (lactate versus a combination of xylose, H_2_, and acetate). We also show that, under certain conditions, production of MCFA provides energetic benefits compared to production of butyrate, thus generating hypotheses for how to control the final products of chain elongation. This knowledge lays a foundation to begin addressing how to engineer and control MCFA-producing microbiomes.

## RESULTS

### Microbiome characterization.

**FIG 1:**
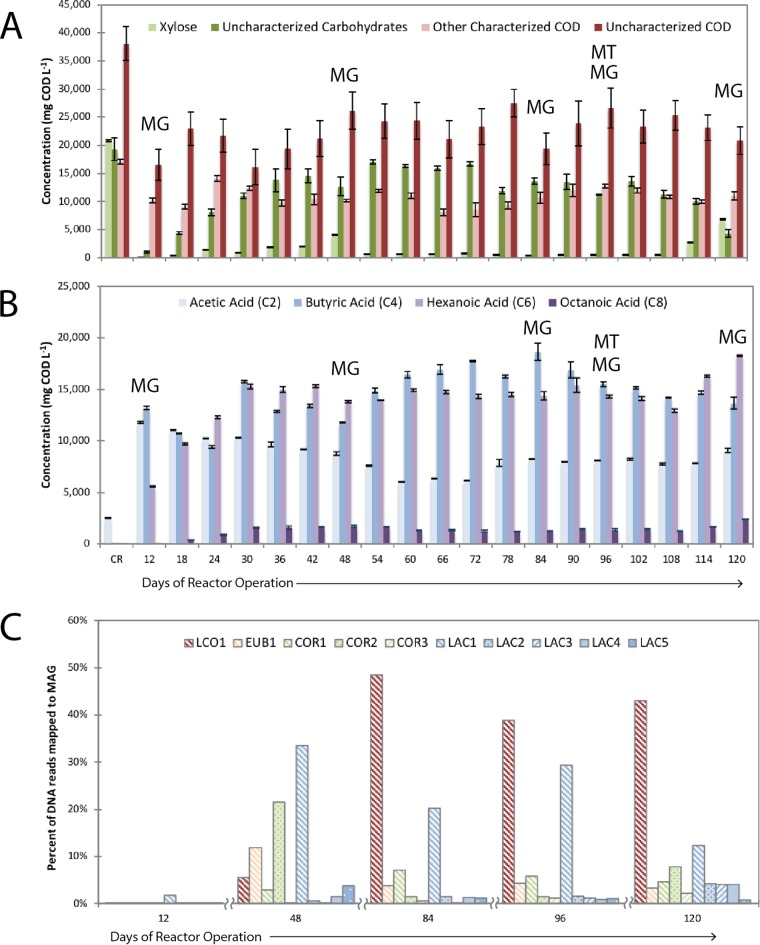
Transformation of materials in lignocellulosic ethanol conversion residues by an anaerobic microbiome and abundance of MAGs. During 120 days of reactor operation, compounds in conversion residues (CR) were converted to medium-chain fatty acids. In panels A and B, the bars in the first set of bars in the figure indicate the concentrations in the feed (CR), whereas the rest of the bars describe concentrations in the reactor. A more detailed description of the operation of this reactor is presented elsewhere (4). Samples were taken for metagenomic (MG) analysis from five time points (day 12, day 48, day 84, day 96, and day 120) and for metatranscriptomic analysis (MT) from one time point (day 96). Overall, the bioreactor transformed xylose, uncharacterized carbohydrates, and uncharacterized COD to acetic (C2), butyric (C4), hexanoic (C6), and octanoic (C8) acids. The microbial community was enriched in 10 MAGs.

We previously described the establishment of a microbiome that produces MCFA in a bioreactor that is continuously fed with the residues from lignocellulosic ethanol production (4). The reactor feed, identified as conversion residues (CR) in Fig. 1, contained high amounts of xylose, carbohydrate oligomers, and uncharacterized organic matter. To gain insight into the microbial activities that were associated with this MCFA-producing microbiome, samples were collected for metagenomic analysis at five different times (days 12, 48, 84, 96, and 120), and RNA was prepared for metatranscriptomic analysis at day 96. At the time of metatranscriptomic sampling, the bioreactor converted 16.5% of the organic matter (measured as chemical oxygen demand [COD]) in conversion residues to C6 and C8. During the period of reactor operation described in the Fig. 1 legend, the bioreactor converted 16.1% ± 3.1% of COD to C6 and C8; therefore, the day 96 data are representative of the overall reactor performance.

**FIG 2:**
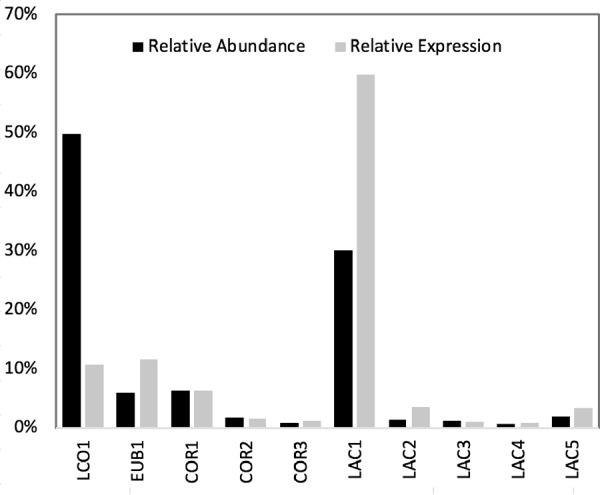
Relative abundance and expression of the 10 most abundant MAGs in the bioreactor at day 96. Relative abundance was determined by mapping DNA sequencing reads to the MAG and normalizing to the length of the MAG genome. Relative transcript abundance (expression) was determined by mapping cDNA sequencing reads to the MAG and normalizing to the length of the MAG genome.

From the metagenomic samples, a total of 219 million DNA reads were assembled and binned, resulting in 37 high-quality (>80% complete, <10% contamination) metagenome-assembled genomes (MAGs) (see Data Set S1 in the supplemental material). In this study, MAGs constitute the collection of genes that were assembled into contigs and represent the population of organisms associated with this collection. For the day 96 sample, 86% of the DNA reads mapped to the 10 most abundant MAGs (Table S1), and each individual MAG mapped to either more than 0.9% of the DNA reads or more than 0.9% of the cDNA reads (Data Set S2). The abundance of the top 10 MAGs was calculated from the percentage of the total DNA reads from each time point mapped to the MAGs (Fig. 1C). For the day 96 sample, relative abundance and expression levels were compared (Fig. 2; see also Data Set S2). The most abundant MAGs included a *Lachnospiraceae* (LCO1; 50%), a *Lactobacillus* (LAC1; 30%), a *Coriobacteriaceae* (COR1; 6.3%), and a *Eubacteriaceae* (EUB1; 6.0%) MAG. Four additional *Lactobacillus* MAGs and two additional *Coriobacteriaceae* MAGs are also predicted to be within the 10 most abundant MAGs (Fig. 2). The other MAGs (Data Set S1) corresponded to *Firmicutes* (17 MAGs), *Actinobacteria* (4 MAGs), *Tenericutes* (3 MAGs), *Bacteroidetes* (2 MAGs), and *Spirochaetes* (1 MAG).

10.1128/mSystems.00221-18.4 DATA SET S1 Summary of all MAGs obtained from metagenomes. This supplement provides the IDs and CheckM results for all 37 of the high-quality (>80% completeness, <10% contamination) MAGs binned from the metagenome coassembly. Download Data Set S1, XLSX file, 0.01 MB. Copyright © 2018 Scarborough et al. 2018 Scarborough et al. This content is distributed under the terms of the Creative Commons Attribution 4.0 International license.

10.1128/mSystems.00221-18.5 DATA SET S2 Read mapping results. This supplement provides the results of mapping DNA and cDNA reads back to the metagenome assembly and individual MAGs analyzed for this study. The relative abundance calculations are also included. Download Data Set S2, XLSX file, 0.01 MB. Copyright © 2018 Scarborough et al. 2018 Scarborough et al. This content is distributed under the terms of the Creative Commons Attribution 4.0 International license.

The metatranscriptome data, obtained from the day 96 sample, contained 87 million cDNA reads. After quality checking and removal of rRNA sequences, 82.6 million predicted transcript reads were used for mapping to MAGs. Of these, 85% of the predicted transcripts (referred to here as transcripts or mRNA) mapped back to the 10 most abundant MAGs (Data Set S2). Relative expression levels were calculated from the total filtered mRNA mapped to the MAGs and normalized to the predicted genome length of these bacteria (Fig. 2). The MAGs with the highest levels of transcripts included LAC1 (60%), EUB1 (12%), LCO1 (11%), and COR1 (6.3%), which also displayed high abundance in the metagenome (Fig. 2). Whereas LCO1 was most abundant on the basis of DNA reads, LAC1 appeared to have the highest activity on the basis of transcript levels.

**FIG 3:**
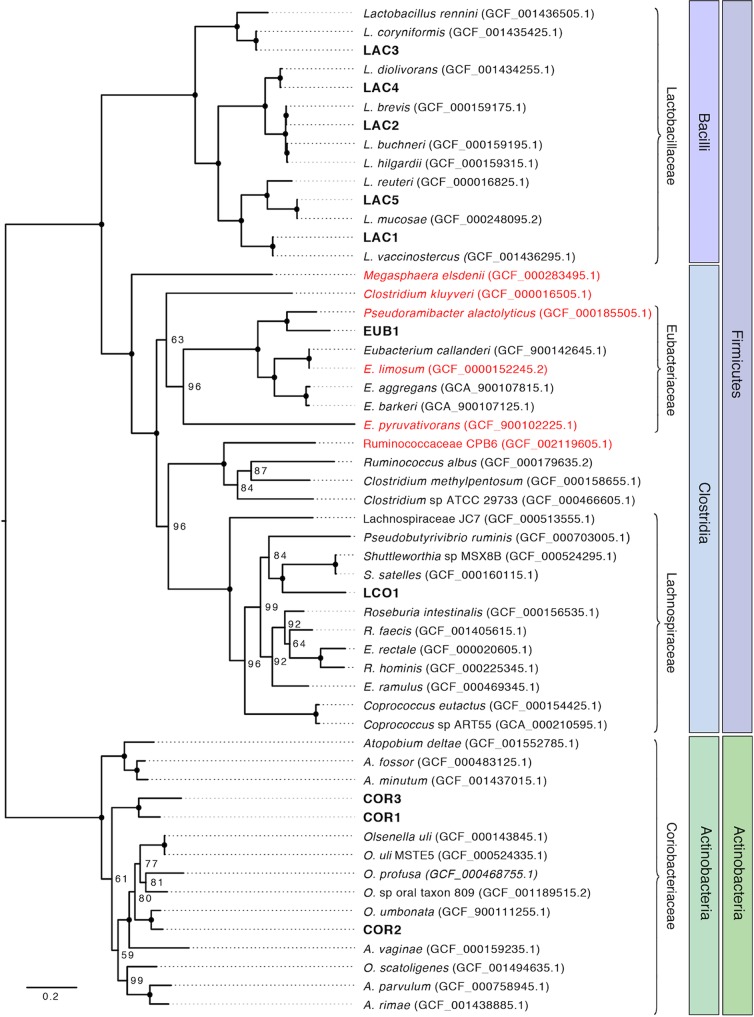
Phylogenetic analysis of 10 MAGs obtained from reactor biomass. Draft genomes from this study are shown in bold text. Red text indicates an organism that has been shown to produce MCFA. National Center for Biotechnology Information assembly accession numbers are shown in parentheses. Node labels represent bootstrap support values, with solid circles representing a bootstrap support value of 100. The phyla and class of genomes are shown in shaded boxes, and families are indicated by brackets.

A phylogenetic tree of the 10 most abundant MAGs was constructed on the basis of concatenated amino acid sequences of 37 single-copy marker genes (Fig. 3). All *Bacilli* MAGs (LAC1, LAC2, LAC3, LAC4, and LAC5) clustered with *Lactobacillus.* Results of average nucleotide identity (ANI) calculations performed with other *Lactobacillus* genomes were above the 95% to 96% ANI level suggested for species demarcation (21), indicating that these MAGs represent strains of established *Lactobacillus* species (Data Set S3). *Clostridia* EUB1 clustered with Pseudoramibacter alactolyticus. The ANI calculations for this MAG with P. alactolyticus and related members of the *Eubacteriaceae* indicated that EUB1 could represent a new genus within the *Eubacterium* (Data Set S3). The EUB1 MAG is likely the same as that of the organism represented by the *Pseudoramibacter* operational taxonomic unit (OTU) in the 16S rRNA-based identification reported earlier (4). The *Clostridia* LCO1 did not cluster with a specific genus; ANIs of the LCO1 MAG and related organisms suggest that this MAG could represent a novel genus within the *Lachnospiraceae* (Data Set S3), while the 16S rRNA-based analysis misclassified it as belonging to the *Roseburia* genus (4). The three *Actinobacteria* MAGs (COR1, COR2, and COR3) clustered within the *Coriobacteriaceae* family. One of these MAGs (COR2) clustered with Olsenella umbonata (22), but the ANI calculation did not support this MAG being a representative of the *Olsenella* genus (Data Set S3). The other two MAGs (COR1 and COR3) formed their own cluster within the *Coriobacteriaceae* but were sufficiently different in ANI calculations to suggest that each represents new genera within the *Coriobacteriaceae*. Phylogenetic classifications of the other MAGs obtained in this study are provided in Data Set S1.

10.1128/mSystems.00221-18.6 DATA SET S3 Average nucleotide identity results. This supplement provides the results of the ANI calculations performed with JSpecies. Download Data Set S3, XLSX file, 0.03 MB. Copyright © 2018 Scarborough et al. 2018 Scarborough et al. This content is distributed under the terms of the Creative Commons Attribution 4.0 International license.

### Genomic predictions of chemical transformations in the microbiome.

A prediction of metabolic networks in the microbiome was performed by analysis of gene annotations for each of the abundant MAGs, whereas expression of the metabolic network was analyzed by mapping mRNA reads to the open reading frames (ORFs) within each of the 10 most abundant bacteria. Metabolic reconstruction was performed with automated prediction algorithms (23) and manual curation, particularly for analysis of proposed sugar utilization, fermentation, and chain elongation pathways (Data Set S4) (1). This analysis identified a set of genes that could be used to model the metabolic potential of the microbiome and also a set of genes with high levels of expression in the metatranscriptome. These gene sets were used to analyze the metabolic potential of the microbiome to (i) degrade complex carbohydrates remaining in ethanol conversion residue; (ii) transform simple sugars into the fermentation products acetate, lactate, and ethanol; and (iii) produce C4, C6, and C8 from sugars and fermentation products. The predictions for each of these processes are summarized below.

10.1128/mSystems.00221-18.7 DATA SET S4 Functional annotations and gene expression. This supplement provides functional annotations of all of the ORFs for the 10 MAGs analyzed for this study. Gene expression results are also included as RPKM values. Download Data Set S4, XLSX file, 2.9 MB. Copyright © 2018 Scarborough et al. 2018 Scarborough et al. This content is distributed under the terms of the Creative Commons Attribution 4.0 International license.

### (i) Degradation of complex carbohydrates.

Carbohydrates represented a large portion of the organic substrates present in the ethanol conversion residue fed to the bioreactor, including uncharacterized carbohydrates. Quantitative analyses indicated that xylose was the most abundant monosaccharide in the residue, accounting for 22% of the organic matter. Glucose was undetected in most samples or was a minor component, and other carbohydrates corresponded to 20% of the organic matter in the residue (see CR bar in Fig. 1). Approximately 40% of the uncharacterized carbohydrates were being degraded at the time that the metatranscriptomic samples were obtained (day 96; Fig. 1).

To investigate the expression of genes related to degradation of complex carbohydrates, we analyzed the predicted MAG ORFs using the carbohydrate-active enzyme (CAZyme) database (24). Production of predicted extracellular enzymes that hydrolyze glycosidic bonds in complex carbohydrates was of particular interest, as these may release sugars that can be subsequently metabolized by community members that do not express complex carbohydrate-degrading enzymes. CELLO subcellular localization software was used to predict whether individual CAZyme proteins were located within the cytoplasm or targeted to the extracellular space (Data Set S5) (25).

10.1128/mSystems.00221-18.8 DATA SET S5 CaZY results. This supplement includes results from annotating ORFs with the CaZY database (http://www.cazy.org). Download Data Set S5, XLSX file, 0.2 MB. Copyright © 2018 Scarborough et al. 2018 Scarborough et al. This content is distributed under the terms of the Creative Commons Attribution 4.0 International license.

This analysis showed that transcripts encoding genes for several types of glycoside hydrolases (GHs) were abundant in several MAGs in the microbiome (see Fig. S1 in the supplemental material). All LAC MAGs expressed genes encoding extracellular CAZymes that cleave glycosidic bonds between hexose and pentose moieties in xylans. In particular, LAC1, LAC2, and LAC4 expressed genes that encode several extracellular exo-β-xylosidases that could remove terminal xylose molecules from xylans present in the conversion residue (GH43 and GH120) (Fig. S1; see also Data Set S5). LAC2 also had high levels of transcripts for an exo-α-L-1,5-arabinanase (GH93), predicted to release other pentose sugars from arabinan, which accounts for 3% of the sugar polymers in switchgrass (26, 27). In addition, the COR1, COR3, and LAC4 members of the community had high transcript levels for three extracellular CAZymes (GH13) that are predicted to degrade a variety of glucans that may be remaining in switchgrass conversion residue (28). In sum, the results of this analysis suggest that at the time of sampling, glucans were degraded by populations represented by *Lactobacillus* and *Coriobacteriaceae* MAGs, where the populations represented by the LAC MAGs may also have had degraded xylans and arabinans. The data further suggest that this microbiome is capable of releasing oligosaccharides and sugar monomers from glucans, xylans, and arabinans, the primary components of switchgrass and other plant biomass. The results also suggest that LCO1 and EUB1 were not participating in complex carbohydrate degradation.

10.1128/mSystems.00221-18.1 FIG S1 Relative expression of extracellular and cytoplasmic glycoside hydrolase enzymes. Circle size indicates the number of ORFs annotated within each bin as the indicated CAZyme family. CAZyme families are grouped according to the target substrate moieties, representing bonds between hexoses only, bonds between hexoses and pentoses, bonds between pentoses only, peptidoglycan-specific moieties, or acid moieties. The relative RPKM (relRPKM) value is normalized to the median RPKM value for the MAG. Gene expression data are presented as log_2_(relRPKM) values. The color intensity gradations represent the relative gene expression levels, with red color intensity indicating expression below median levels and blue intensity indicating expression above median levels. The expression level represents the highest relative expression level for an individual gene within the indicated family. Descriptions of each family are available through the CAZY database (http://www.cazy.org/Glycoside-Hydrolases.html). Download FIG S1, TIF file, 2.6 MB. Copyright © 2018 Scarborough et al. 2018 Scarborough et al. This content is distributed under the terms of the Creative Commons Attribution 4.0 International license.

Bacterial oligosaccharide hydrolysis can also occur in the cytoplasm. All MAGs in this microbiome contained predicted cytoplasmic GH13 enzymes, which are known to degrade hexose oligosaccharides. The microbiome also contained abundant transcripts for genes encoding predicted cytoplasmic CAZYmes that degrade maltose (GH4 and GH65), a glucose dimer that may result from extracellular breakdown of glucans (Fig. S1). Transcripts encoding known or predicted cytoplasmic β-glucosidases (GH1 and GH3) and β-galactosidases (GH2) were found across the MAGs (Fig. S1). In addition, transcripts that encode β-xylosidases (GH1 and GH3) and α-l-arabinofuranosidases (GH2) were found in all the LAC MAGs except LAC3 (Fig. S1). On the basis of the metatranscriptomic analysis, other cytoplasmic CAZymes predicted to hydrolyze pentose-containing oligosaccharides are predicted to be expressed by the LAC1, LAC2, LAC4, and LAC5 members of this microbiome (Fig. S1).

### (ii) Transport and production of simple fermentation products from sugars.

Simple sugars are abundant in ethanol conversion residue and are produced during complex carbohydrate hydrolysis. Sugars are therefore expected to be a major substrate for the microbiome. Despite the use of a yeast strain that was engineered for improved xylose utilization in the ethanol fermentation, xylose was the major abundant monosaccharide present in the remaining conversion residue (CR; Fig. 1). As discussed above, the relative transcript levels of genes encoding extracellular GHs (Fig. S1) by several MAGs in the microbiome suggest that additional pentoses and hexoses may be released through degradation of complex carbohydrates.

**FIG 4:**
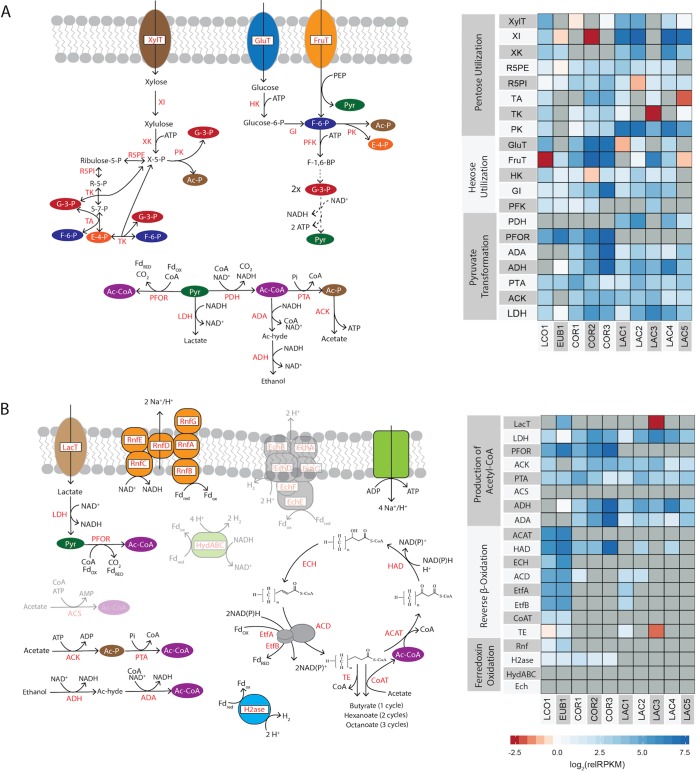
Relative expression of genes involved in the conversion of xylose and lactate to MCFA. Expression levels of key enzymes involved in (A) utilization of xylose and (B) acyl chain elongation are indicated. Dashed lines represent the existence of multiple enzyme reactions between the indicated molecules. The relative RPKM (relRPKM) values are normalized to the median RPKM for the MAG. Gene expression data are presented as log_2_(relRPKM) values. The color intensities of the heatmaps represent the relative gene expression levels, with red color intensity indicating expression below median levels and blue intensity indicating expression above median levels. Gray indicates that the gene is absent from the genome. For genes that are not predicted to be expressed by any MAGs, the associated enzyme product is grayed out in the pathway figure. Key pathway intermediates include xylulose, xylulose-5-phosphate (X-5-P), ribose-5-phosphate (R-5-P), sedoheptulose-7-phosphate (S-7-P), erythyrose-4-phosphate (E-4-P), glyceraldehyde-3-phosphate (G-3-P), fructose-6-phosphate (F-6-P), acetate (Ac), acetyl-phosphate (Ac-P), acetyl-CoA (Ac-CoA), lactate, and ethanol. Enzyme abbreviations are as follows. (A) XylT = xylose transporter, XI = xylose isomerase (EC 5.3.1.5); XK = xylulose kinase (EC 2.7.1.17), R5PE = ribulose-5-phosphate epimerase (EC 5.1.3.1), R5PI = ribose-5-phosphate isomerase (EC 5.3.1.6), TA = transaldolase (EC 2.2.1.2), TK = transketolase (EC 2.2.1.1), PK = d-xyulose 5-phosphate/d-fructose 6-phosphate phosphoketolase (EC 4.1.2.9), GluT = glucose transporter, FruT = fructose PTS transporter, HK = hexokinase (EC 2.7.1.1, EC 2.7.1.2), G6PI = glucose-6-phoshphate isomerase (EC 5.3.1.9), PFK = phosphofructokinase (EC 2.7.1.11), PDH = pyruvate dehydrogenase complex (EC 1.2.4.1, EC 2.3.1.12, EC 1.8.1.4), PFOR = pyruvate flavodoxin oxidoreductase (EC 1.2.7.-), ADA = acetaldehyde dehydrogenase (EC 1.2.1.10), AD = alcohol dehydrogenase (EC 1.1.1.1), PTA = phosphate acetyltransferase (EC 2.3.1.8), ACK = acetate kinase (EC 2.7.2.1), and LDH = lactate dehydrogenase (EC 1.1.1.27). (B) LacT = lactate permease, ACS = acetyl-CoA synthetase (EC 6.2.1.1), ACAT = acetyl-CoA C-acyltransferase (EC 2.3.1.16, EC 2.3.1.9), HAD = 3-hydroxyacyl-CoA dehydrogenase (EC 1.1.1.157, 1.1.1.35), ECH = enoyl-CoA hydratase (EC 4.2.1.55, EC 4.2.1.17), ACD = acyl-CoA Dehydrogenase (EC 1.3.99.2, EC 1.3.99.-), EtfA = electron transfer flavoprotein A, EtfB = electron transfer flavoprotein B, TE = thioesterase (EC 3.1.2.20), CoAT = 4-hydroxybutyrate CoA transferase (EC 2.8.3.-), Rnf = ferredoxin–NAD^+^ oxidoreductase–Na+ translocating (EC 1.18.1.8), H2ase = ferredoxin hydrogenase (EC 1.12.7.2), HydABC = bifurcating [Fe-Fe] hydorgenase (EC 1.12.1.4), Ech = energy-conserving hydrogenase (EchABCDEF).

We therefore analyzed the genomic potential of the community to transport sugars and to metabolize them to fermentation products, particularly the known MCFA precursors lactate, acetate, and ethanol. To investigate the ability of the community to transport sugars, MAG ORFs were annotated using the Transporter Classification Database (Data Set S6). Expression of genes associated with the pentose phosphate pathway, phosphoketolase (PK) pathways, and glycolysis (Fig. 4A) was analyzed to predict the potential for sugar metabolism within individual MAGs.

10.1128/mSystems.00221-18.9 DATA SET S6 Transporter database. This supplement includes results from annotating ORFs with the transporter database (http://www.tcdb.org). Download Data Set S6, XLSX file, 0.6 MB. Copyright © 2018 Scarborough et al. 2018 Scarborough et al. This content is distributed under the terms of the Creative Commons Attribution 4.0 International license.

This analysis found that transcripts from genes encoding predicted carbohydrate transporters were among the most highly abundant mRNAs across the microbiome, accounting for 5.8% of the total transcripts. These putative transporters belonged to a variety of families, including many associated with the ATP-binding cassette (ABC) superfamily and with the phosphotransferase system (PTS) family (Fig. S2). LCO1, LAC1, LAC2, and LAC3 are predicted to contain xylose transporters (XylT) (Fig. 4A), while glucose (GluT), fructose (FruT), and other hexose transporters were expressed across the LAC, COR, and LCO MAGs (Fig. 4A; see also Fig. S2). EUB1 carried only transcripts encoding carbohydrate transporters for uptake of fructose and sucrose (Fig. S2). Overall, this analysis predicts that all MAGs have the potential to transport hexose sugars into the cell, while the gene expression patterns observed for the LCO1 and the *Lactobacillus* MAGs (excluding LAC3) predicted that they played a major role in pentose utilization in this microbiome at the time of sampling.

10.1128/mSystems.00221-18.2 FIG S2 Expression of known or predicted transporters by the 10 most abundant microbiome members. Transporters were annotated by use of the Transporter Classification Database. (A) The number of genes and relative expression for each analyzed MAG are shown for Transporter Database families. Circle size indicates the number of ORFs annotated within each bin as the indicated CAZY class or family. The color intensity represents the relative gene expression according to the shown scale. The represented expression is the highest relative expression for an individual gene within the indicated transporter family. (B) The expected substrates, representing a pentose (5C-Mono), a pentose-containing oligosaccharide (5C-Oligo), a hexose (6C-mono), a hexose disaccharide (6C-Di), or a hexose-containing oligosaccharide (6C-Oligo), are indicated by orange squares. The color intensity represents the relative gene expression according to the shown scale. The transporter family identifiers (IDs) and descriptions are shown. More-detailed descriptions of transporters are available from the Transporter Database (http://www.tcdb.org). Download FIG S2, TIF file, 0.4 MB. Copyright © 2018 Scarborough et al. 2018 Scarborough et al. This content is distributed under the terms of the Creative Commons Attribution 4.0 International license.

We also analyzed the metatranscriptomic data to investigate potential routes for sugar metabolism. Once transported to the cytoplasm, glucose can be phosphorylated with hexokinase (HK) and converted to fructose-6-phosphate (F-6-P) by glucose-6-phosphate isomerase (GI). Transcripts encoding predicted HK and GI enzymes were abundant for all MAGs within the microbiome (Fig. 4A), except LAC5, for which the assembly did not show homologues of these proteins. Fructose utilization started with phosphorylation during transport (Fig. 4A). Fructose-6-phosphate (F-6-P) either is phosphorylated to fructose-1,6-bisphosphate (F-1,6-BP) by phosphofructokinase (PFK) in glycolysis or is cleaved to acetyl-P (Ac-P) and erythrose-4-P (E-4-P) by phosphoketolase (PK). While LAC1, LAC2, LAC4, LAC5, and COR3 all lack homologues of genes encoding PFK (a highly conserved glycolysis enzyme known to be a major target for regulatory control in hexose utilization) (29), they all contain transcripts for homologues of PK (Fig. 4A). In sum, these analyses suggest that all of the abundant MAGs in this microbiome can utilize hexoses that may be produced during hydrolysis of complex oligosaccharides.

Transcripts predicted to encode enzymes to convert xylose to xylulose-5-phosphate, xylose isomerase (XI), and xylulose kinase (XK) (30) were abundant in most of the *Lactobacillus* MAGs and LCO1 and either were absent or showed very low abundance in LAC3, EUB1, and the COR MAGs (Fig. 4A). Once produced, xylulose-5-P can be degraded through either the phosphoketolase pathway or the pentose phosphate pathway. Transcripts from a gene predicted to encode the diagnostic enzyme of the phosphoketolase pathway, phosphoketolase (PK), which splits xylulose-5-P (X-5-P) into acetyl-P (Ac-P) and glyceraldehyde-3-P (G-3-P), were among the most abundant mRNAs in the *Lactobacillus* MAGs and were also present at high levels in LCO1, accounting for 1.5% of the total transcripts (Fig. 4A; see also Data Set S4). LCO1 and LAC1 also contained transcripts from homologues of all of the genes needed for the pentose phosphate pathway (R5PE, R5PI, TA, and TK [Fig. 4A]). Overall, this analysis predicted that multiple routes of pentose utilization could be utilized by the MAGs in this microbiome.

The predicted routes for both hexose and xylose metabolism in this microbiome lead to pyruvate production (Fig. 4A), so we also analyzed how this and other fermentation products might lead to MCFA production in this community. All MAGs contained transcripts encoding lactate dehydrogenase homologues (LDH) (Fig. 4A), an enzyme which reduces pyruvate to lactate. Transcript analysis also predicts that all of the MAGs (except LAC3) can oxidize pyruvate to acetyl-CoA, utilizing either pyruvate dehydrogenase (PDH) or pyruvate flavodoxin oxidoreductase (PFOR) (Fig. 4A). All MAGs (except EUB1) contain transcripts encoding homologues of acetate kinase (ACK), which converts acetyl-phosphate (Ac-P) to acetate while producing ATP (Fig. 4A). On the basis of predictions of the gene expression data, the COR and LAC MAGs are also able to convert acetyl-CoA (Ac-CoA) to ethanol with aldehyde dehydrogenase (ADA) and alcohol dehydrogenase (ADH). In summary, analysis of the gene expression patterns in the conversion residue microbiome predicts that the MAGs in the LCO, LAC, and COR ferment sugars to acetate and lactate, while the LAC and COR members produce ethanol as an additional fermentation product.

### (iii) Elongation of fermentation products to MCFAs.

On the basis of the findings reported above, we analyzed the microbiome gene expression data to predict which members of the microbiome had the potential for conversion of predicted fermentation products to MCFA. The *Clostridia* (LCO1 and EUB1) are the only MAGs that contained genes encoding homologues of enzymes known to catalyze chain elongation reactions in the reverse β-oxidation pathway (Fig. 4B). Thus, the subsequent analysis is based on the prediction that only LCO1 and EUB1 are the major producers of MCFA in this microbiome. Furthermore, on the basis of the analysis of sugar utilization above, we suggest that LCO1 is the only microorganism in the community that can directly utilize sugars for MCFA production.

Acetate, lactate, and ethanol are all fermentation products that would require transformation to acetyl-CoA before being used as a substrate for elongation by the reverse β-oxidation pathway. Acetate could be converted to acetyl-CoA, utilizing ATP via acetyl-CoA synthase (ACS) or the ACK and phosphate acetyltransferase (PTA) route (Fig. 4B). Alternatively, acetate can be converted to acetyl-CoA with a CoA transferase (CoAT) which transfers a CoA from one carboxylic acid to another (e.g., from butyryl-CoA to acetate, producing butyrate and acetyl-CoA) (Fig. 4B). Genes encoding homologues of ACS and ACK were not found in EUB1, but LCO1 contained abundant transcripts that encoded homologues of both ACK and PTA (Fig. 4A). Both MAGs also contained transcripts predicted to encode CoAT enzymes (Fig. 4B). Taking the data together, this analysis suggests that acetate may be used as a substrate for MCFA production by LCO1 and EUB1.

Lactate has been proposed as a key intermediate in other microbiomes producing MCFA (12). While transcripts encoding genes for lactate production were abundant in the microbiome (Fig. 4A), lactate did not accumulate to detectable levels during steady operation but transiently accumulated when the bioreactor received a higher load of conversion residue (4). Transcripts for a gene encoding a predicted lactate transporter (LacT) were abundant in EUB1. In addition, the assembly of LCO1 did not reveal the presence of lactate transporter genes in this MAG, suggesting that only EUB1 can utilize the lactate produced by other MAGs. Neither EUB1 nor LCO1 accumulated transcripts encoding a predicted ADA homologue, which would be required for conversion of acetaldehyde to acetyl-CoA during utilization of ethanol (Fig. 4B). This indicates that if ethanol is produced in this microbiome, it is not used as a significant substrate for MCFA production. Moreover, since ethanol did not accumulate in the reactor either during steady-state operation (Fig. 1) or after addition of a high load of conversion residue (4), we suggest that ethanol is not a substrate for MCFA production in this microbiome. Rather, on the basis of the predicted activity of LAC and COR MAGs producing lactate and that of EUB1 consuming lactate, we suggest that lactate is a key fermentation intermediate for MCFA production.

Within the reverse β-oxidation pathway (Fig. 4B), a key enzyme is an electron-bifurcating acyl-CoA dehydrogenase (ACD) containing two electron transfer flavoproteins (EtfA and EtfB) that pass electrons from NADH to ferredoxin (Fig. 4B) (31). This electron-bifurcating complex has been recognized as a key energy-conserving mechanism in strictly anaerobic bacteria and archaea (17, 31) and studied in detail in butyrate-producing anaerobes (32, 33). Transcripts for genes encoding the acyl-CoA dehydrogenase complex (ACD) and homologues (EtfA and EtfB) were abundant in both LCO1 and EUB1, as were transcripts for other genes predicted to be involved in this pathway (Fig. 4B). Chain elongation by the reverse β-oxidation pathway conserves energy by increasing the ratio of reduced ferredoxin (a highly electropositive electron carrier) to the less electropositive NADH (1). In organisms that use this pathway, oxidation of ferredoxin by the Rnf complex generates an ion motive force, and ATP synthase utilizes the ion motive force to produce ATP (17). We found that transcripts for genes encoding homologues of all six subunits of the Rnf complex were abundant in both EUB1 and LCO1 (RnfABCDEG; Fig. 4B). To maintain cytoplasmic redox balance, reduced ferredoxin could transfer electrons to H^+^ via hydrogenase, generating H_2_. LCO1 and EUB1, along with the COR MAGs, contained abundant transcripts for genes predicted to produce ferredoxin hydrogenase (H2ase; Fig. 4B), supporting the hypothesis that H_2_ production plays a role within this MCFA-producing microbiome. We also looked for two additional hydrogenases known to conserve energy either through the translocation of protons (EchABCDEF; Fig. 4B) or by electron confurcation, utilizing electrons from both NADH and reduced ferredoxin (HydABC; Fig. 4B) (17). It does not appear that these systems play a major role in H_2_ production in this microbiome since none of the MAGs contained genes encoding homologues of the known components for either of these enzyme complexes (Fig. 4B).

### Thermodynamic analysis of MCFA production in the microbiome.

The analysis described above predicted several potential routes for MCFA production by LCO1 and EUB1 in this microbiome. To evaluate the implications of these potential chain elongation routes, we used thermodynamic analysis to investigate the energetics of the predicted transformations. For this, we reconstructed metabolic pathways for xylose and lactate conversion and determined ATP yields on the basis of the data obtained from gene expression analyses (Tables 1 and 2; see also Data Set S7). Metabolic reconstructions indicated that xylose (Table 1) and lactate (Table 2) are major substrates for synthesis of C4, C6, and C8 products. In addition, both LCO1 and EUB1 have the potential to use a CoAT or a thioesterase (TE) as the terminal enzyme of the reverse β-oxidation pathway (Fig. 4B), so we considered both possibilities in the thermodynamic analysis. We used these reconstructions to calculate the free energy changes of the overall biochemical reactions by assuming an intracellular pH of 7.0, a temperature of 35°C, and H_2_ partial pressures of 1.0 × 10^−6^, 1.0, and 6.8 atm for low, standard, and high H_2_ partial pressure, respectively. The low value represents the approximate concentration of H_2_ in water that is in equilibrium with the atmosphere and the high value represents an expected maximum in a pressurized mixed-culture fermentation system (34). We also compared the efficiency of ATP production to an expected maximum yield of 1 ATP per −60 kJ energy generated by the overall chemical transformation (17).

**TABLE 1:** Thermodynamics of biochemical reactions involved in conversion of xylose to butyrate, hexanoate, and octanoate^*a*^

Equation no.	Equation	Associated MAG(s)	ΔG per mol substrate^*b*^ (kJ mol^−1^)			*Y*_ATP_ (mol ATP mol^−1^ substrate)^*b*^^,^^*c*^	ΔG^0'^ available per ATP produced (kJ mol^−1^ ATP)	Terminal enzyme
			P_H2_ = 10^–6^ atm	P_H2_ = 1 atm	P_H2_ = 6.8 atm			
Xylose simple fermentation								
1	3 C_5_H_10_O_5_ → 5 C_3_H_5_O_3_^-^ + 5 H^+^	LAC1, LAC2, LAC4, LAC5	−174	−174	−174	1.67	−104 to −104	
2	3 C_5_H_10_O_5_ → 3 C_3_H_5_O_3_^-^ + 3 C_2_H_3_O_2_^-^ + 6 H^+^	LAC1, LAC2, LAC4, LAC5	−214	−214	−214	2.00	−107 to −107	

Xylose elongation								
3	3 C_5_H_10_O_5_ → 3 C_4_H_7_O_2_^-^ + 3 CO_2_ + 3 H_2_O + 3 H^+^	LCO1	−264	−264	−264	3.00	−88 to −88	CoAT
4	3 C_5_H_10_O_5_ → 1 C_6_H_11_O_2_^-^ +3 C_2_H_3_O_2_^-^ + 3 CO_2_ + 4 H^+^ + 2 H_2_	LCO1	−272	−248	−245	2.83	−87 to −96	CoAT
5	3 C_5_H_10_O_5_ → 1 C_8_H_15_O_2_^-^ + 2 C_2_H_3_O_2_^-^ + 3 CO_2_ + 3 H_2_O + 3 H^+^	LCO1	−265	−265	−265	3.00	−88 to −88	CoAT
6	2 C_5_H_10_O_5_ → 1 C_4_H_7_O_2_^-^ + 2 C_2_H_3_O_2_^-^ + 2 CO_2_ + 3 H^+^+ 2 H_2_	LCO1	−276	−240	−235	2.25	−105 to −123	TE
7	3 C_5_H_10_O_5_ → 1 C_6_H_11_O_2_^-^ + 3 C_2_H_3_O_2_^-^ + 3 CO_2_ + 1 H_2_O + 4 H^+^ + 2 H_2_	LCO1	−272	−248	−245	2.50	−98 to −109	TE
8	4 C_5_H_10_O_5_ → 1 C_8_H_15_O_2_^-^ + 4 C_2_H_3_O_2_^-^ + 4 CO_2_ + 2 H_2_O + 5 H^+^+ 2 H_2_	LCO1	−270	−253	−250	2.63	−95 to −103	TE

Xylose and C2/C4/C6^*d*^ elongation								
9	1 C_5_H_10_O_5_ + 2 C_2_H_3_O_2_^-^ + 2 H_2_ → 2 C_4_H_5_O_2_^-^ + 1 CO_2_ + 3 H_2_O	LCO1	−240	−311	−320	3.50	−69 to −92	CoAT
10	1 C_5_H_10_O_5_ + 1 C_2_H_3_O_2_^-^ + 2 H_2_ → 1 C_6_H_11_O_2_^-^ + 1 CO_2_ + 3 H_2_O	LCO1	−240	−311	−320	3.50	−69 to −92	CoAT
11	1 C_5_H_10_O_5_ + 1 C_4_H_7_O_2_^-^ + 2 H_2_ → 1 C_8_H_15_O_2_^-^ + 1 CO_2_ + 3 H_2_O	LCO1	−264	−264	−264	3.50	−75 to −75	CoAT
12	1 C_5_H_10_O_5_ + 1 C_4_H_7_O_2_^-^ → 1 C_6_H_11_O_2_^-^ + 1 C_2_H_3_O_2_^-^ + 1 CO_2_ + 1 H_2_O + 1 H^+^	LCO1	−243	−314	−324	3.00	−81 to −108	CoAT
13	1 C_5_H_10_O_5_ + 1 C_6_H_11_O_2_^-^ → 1 C_8_H_15_O_2_^-^ + 1 C_2_H_3_O_2_^-^ + 1 CO_2_ + 1 H_2_O + 1 H^+^	LCO1	−267	−267	−267	3.00	−89 to −89	CoAT
14	1 C_5_H_10_O_5_ + 2 C_2_H_3_O_2_^-^ + 2 H_2_ → 2 C_4_H_5_O_2_^-^ + 1 CO_2_ + 3 H_2_O	LCO1	−240	−311	−320	1.50	−160 to −214	TE
15	1 C_5_H_10_O_5_ + 1 C_2_H_3_O_2_^-^ + 2 H_2_ → 1 C_6_H_11_O_2_^-^ + 1 CO_2_ + 3 H_2_O	LCO1	−240	−311	−320	2.50	−96 to −128	TE
16	1 C_5_H_10_O_5_ + 1 C_4_H_7_O_2_^-^ + 2 H_2_ → 1 C_8_H_15_O_2_^-^ + 1 CO_2_ + 3 H_2_O	LCO1	−264	−264	−264	2.50	−105 to −105	TE
17	1 C_5_H_10_O_5_ + 1 C_4_H_7_O_2_^-^ → 1 C_6_H_11_O_2_^-^ + 1 C_2_H_3_O_2_^-^ + 1 CO_2_ + 1 H_2_O + 1 H^+^	LCO1	−243	−314	−324	2.00	−122 to −162	TE
18	1 C_5_H_10_O_5_ + 1 C_6_H_11_O_2_^-^ → 1 C_8_H_15_O_2_^-^ + 1 C_2_H_3_O_2_^-^ + 1 CO_2_ + 1 H_2_O + 1 H^+^	LCO1	−267	−267	−267	2.00	−133 to −133	TE

a Free energies of formation for all chemical compounds were obtained from Kbase (www.kbase.us). The ATP yield (*Y*_ATP_) was determined on the basis of biochemical models presented in Data Set S7 and is indicated as moles of ATP produced per mole of xylose consumed. The terminal enzyme of reverse β-oxidation, i.e., either a CoA transferase (CoAT) or thioesterase (TE), is also indicated.

b ΔG values and expected ATP yields are normalized to moles of xylose, moles of lactate, or moles of glycerol.

c The pathway reconstructions shown in Data Set S7 were used to determine the expected ATP yields.

d These scenarios considered coutilization of xylose and acetate (C2), butyrate (C4), or hexanoate (C6).

**TABLE 2:** Thermodynamics of biochemical reactions involved in conversion of lactate to butyrate, hexanoate, and octanoate^*a*^

Equation no.	Equation	Associated MAG(s)	ΔG per mol substrate^*b*^ (kJ mol^−1^)			*Y*_ATP_ (mol ATP mol^−1^ substrate)^*b*^^,^^*c*^	ΔG^0'^ available per ATP produced^*d*^ (kJ mol^−1^ ATP)	Terminal enzyme
			P_H2_ = 10^−6^ atm	P_H2_ = 1 atm	P_H2_ = 6.8 atm			
Lactate elongation								
19	2 C_3_H_5_O_3_^-^ + 1 H^+^ → 1 C_4_H_7_O_2_^-^ + 2 CO_2_ + 2 H_2_	EUB1	−62	−26	−21	0.75	**−29** to −82	CoAT
20	3 C_3_H_5_O_3_^-^ + 2 H^+^ → 1 C_6_H_11_O_2_^-^ + 3 CO_2_ + 2 H_2_ + 1 H_2_O	EUB1	−58	−34	−31	0.83	**−37** to −70	CoAT
21	4 C_3_H_5_O_3_^-^ + 3 H^+^ → 1 C_8_H_15_O_2_^-^ + 4 CO_2_ + 2 H_2_ + 2 H_2_O	EUB1	−57	−39	−36	0.88	**−41** to −64	CoAT
22	2 C_3_H_5_O_3_^-^ + 1 H^+^ → 1 C_4_H_7_O_2_^-^ + 2 CO_2_ + 2 H_2_	EUB1	−62	−26	−21	0.25	−86 to −247	TE
23	3 C_3_H_5_O_3_^-^ + 2 H^+^ → 1 C_6_H_11_O_2_^-^ + 3 CO_2_ + 2 H_2_ + 1 H_2_O	EUB1	−58	−34	−31	0.50	−62 to −116	TE
24	4 C_3_H_5_O_3_^-^ + 3 H^+^ → 1 C_8_H_15_O_2_^-^ + 4 CO_2_ + 2 H_2_ + 2 H_2_O	EUB1	−57	−39	−36	0.63	−58 to −90	TE

Lactate and C2/C4/C6^*g*^ elongation								
25	1 C_3_H_5_O_3_^-^ + 1 C_2_H_3_O_2_^-^ + 1 H^+^ → 1 C_4_H_7_O_2_^-^ + 1 CO_2_ + 1 H_2_O	EUB1	−50	−50	−50	1.00	**−50 to −50**	CoAT
26	2 C_3_H_5_O_3_^-^ + 1 C_2_H_3_O_2_^-^ + 2 H^+^ → 1 C_6_H_11_O_2_^-^ + 2 CO_2_ + 2 H_2_O	EUB1	−50	−50	−50	1.00	**−50 to −50**	CoAT
27	3 C_3_H_5_O_3_^-^ + 1 C_2_H_3_O_2_^-^ + 3 H^+^ → 1 C_8_H_15_O_2_^-^ + 3 CO_2_ + 3 H_2_O	EUB1	−51	−51	−51	1.00	**−51 to −51**	CoAT
28	1 C_3_H_5_O_3_^-^ + 1 C_4_H_7_O_2_^-^ + 1 H^+^ → 1 C_6_H_11_O_2_^-^ + 1 CO_2_ + 1 H_2_O	EUB1	−50	−50	−50	1.00	**−50 to −50**	CoAT
29	2 C_3_H_5_O_3_^-^ + 1 C_4_H_7_O_2_^-^ + 2 H^+^ → 1 C_8_H_15_O_2_^-^ + 2 CO_2_ + 2 H_2_O	EUB1	−51	−51	−51	1.00	**−51 to −51**	CoAT
30	1 C_3_H_5_O_3_^-^ + 1 C_6_H_11_O_2_^-^ + 1 H^+^ → 1 C_8_H_15_O_2_^-^ + 1 CO_2_ + 1 H_2_O	EUB1	−53	−53	−53	1.00	**−53 to −53**	CoAT
31	1 C_3_H_5_O_3_^-^ + 1 C_2_H_3_O_2_^-^ + 1 H^+^ → 1 C_4_H_7_O_2_^-^ + 1 CO_2_ + 1 H_2_O	None^*f*^	−50	−50	−50	0.00	NA^*f*^	TE
32	2 C_3_H_5_O_3_^-^ + 1 C_2_H_3_O_2_^-^ + 2 H^+^ → 1 C_6_H_11_O_2_^-^ + 2 CO_2_ + 2 H_2_O	None^*e*^	−50	−50	−50	0.50	−100 to −100	TE
33	3 C_3_H_5_O_3_^-^ + 1 C_2_H_3_O_2_^-^ + 3 H^+^ → 1 C_8_H_15_O_2_^-^ + 3 CO_2_ + 3 H_2_O	None^*e*^	−51	−51	−51	0.67	−76 to −76	TE
34	1 C_3_H_5_O_3_^-^ + 1 C_4_H_7_O_2_^-^ + 1 H^+^ → 1 C_6_H_11_O_2_^-^ + 1 CO_2_ + 1 H_2_O	None^*e*^	−50	−50	−50	0.00	NA^*f*^	TE
35	2 C_3_H_5_O_3_^-^ + 1 C_4_H_7_O_2_^-^ + 2 H^+^ → 1 C_8_H_15_O_2_^-^ + 2 CO_2_ + 2 H_2_O	None^*e*^	−51	−51	−51	0.50	−103 to −103	TE
36	1 C_3_H_5_O_3_^-^ + 1 C_6_H_11_O_2_^-^ + 1 H^+^ → 1 C_8_H_15_O_2_^-^ + 1 CO_2_ + 1 H_2_O	None^*f*^	−53	−53	−53	0.00	NA^*f*^	TE

a Free energies of formation for all chemical compounds were obtained from Kbase (www.kbase.us). The ATP yield was determined on the basis of biochemical models presented in Data Set S7 and is indicated as moles of ATP produced per mole of lactate consumed. The terminal enzyme of reverse β-oxidation, i.e., either a CoA transferase (CoAT) or thioesterase (TE), is also indicated.

b ΔG values and expected ATP yields are normalized to moles of xylose, moles of lactate, or moles of glycerol.

c Pathway reconstructions shown in Data Set S7 were used to determine the expected ATP yields.

d The minimum expected level of ΔG^0^' per mole of ATP produced is −60 kJ. Values below this are indicated by bold text and indicate that the predicted ATP yield exceeds what is physiologically feasible.

e The proposed model requires acetate kinase either for incorporation of a carboxylate or for ATP generation from acetyl-CoA. EUB1 is not predicted to produce this enzyme.

f NA, no net ATP production is predicted for this model.

g These scenarios considered coutilization of lactate and acetate (C2), butyrate (C4), or hexanoate (C6).

10.1128/mSystems.00221-18.10 DATA SET S7 Thermodynamic models. This supplement includes 36 pathway diagrams used to calculate reaction thermodynamics and ATP yields in Tables 1 and 2. Download Data Set S7, PDF file, 13.4 MB. Copyright © 2018 Scarborough et al. 2018 Scarborough et al. This content is distributed under the terms of the Creative Commons Attribution 4.0 International license.

The use of xylose as the substrate (Table 1, equations 3 to 8) is possible for LCO1 but not EUB1, since the latter MAG lacks genes to transport and activate xylose to xylulose-5-P (XylT, XI, and XK; Fig. 4A). Our analysis suggested that, with a pathway containing a terminal CoAT enzyme, the ATP yield (quantified as moles of ATP per mole of xylose) does not increase if longer-chain MCFA are produced. However, when TE was used for the terminal step of reverse β-oxidation (Table 1, equations 6 to 8), the overall ATP yield was lower but increased with increasing product length, and C8 production provided a 17% increase in ATP yield versus production of C4. This suggests that LCO1 derives no energetic benefit for producing C6 or C8 solely from xylose unless TE is used as the terminal enzyme of reverse β-oxidation. Additionally, the higher ATP yield of xylose conversion to C4 (Table 1, equations 3 and 6) than was seen with xylose conversion to lactate and acetate by other members of the microbiome (Table 1, equation 2) may explain why LCO1 reached higher abundance in the microbiome than was reached by the other, less abundant MAGs (LAC) that are predicted to ferment xylose to lactate and acetate (Fig. 2). In production of C4 and C8, no H_2_ is predicted to be formed if a CoAT is utilized (Table 1, equations 3 and 5), whereas H_2_ production is predicted when C6 is produced (Table 1, equation 4). On the other hand, if a TE terminal enzyme is utilized for the reverse β-oxidation, H_2_ is predicted to be produced for all carboxylic acid products.

Additional metabolic reconstructions analyzed the coutilization of xylose with a monocarboxylic acid (Table 1, equations 9 to 18). This analysis predicted that cometabolism of these substrates could provide an energetic advantage (i.e., higher moles of ATP per mole of xylose) if H_2_ were utilized as an electron donor. This suggests that H_2_, produced by either EUB1 or COR MAGs (H2ase; Fig. 4B), can be utilized by LCO1 to support MCFA production. When TE is used as the terminal enzyme of reverse β-oxidation (Table 1, equations 14 to 18), there is no increase in ATP yield versus utilization of xylose as the sole carbon source (Table 1, equations 6 to 8).

We also modeled MCFA production from lactate by EUB1, since the gene expression data suggested that EUB1 could transform lactate to MCFA. In models utilizing CoAT (Table 2, equations 19 to 21) as a final step in MCFA production, the ATP yield increases as longer-chain MCFA are produced, but the free energy released is near the expected limit for ATP production (17) under conditions of low H_2_ partial pressure and below this limit at high H_2_ partial pressures (Table 2). If TE is utilized as a final step in MCFA production by EUB1 (Table 2, equations 22 to 24), lower ATP yields are predicted, and in that case the production of longer-chain MCFA has a more pronounced effect on the ATP generated per mole of lactate consumed. For instance, production of C6 results in a 100% increase in the ATP yield compared to producing C4. However, each elongation step reduces the amount of energy released per mole of ATP produced, such that production of C8 from lactate results in the release of −58 kJ per ATP produced under high-H_2_ conditions, which is near the expected limits for a cell to conserve chemical energy as ATP. Overall, the thermodynamic analysis does not unequivocally predict which terminal enzyme might be energetically more advantageous for MCFA production from lactate. While using TE would result in more-favorable free energy release than using CoAT, the predicted ATP yields are lower with TE than with CoAT. We also note that although CoAT transcript abundance was higher than TE transcript abundance (Fig. 4B), expression alone cannot be used as a predictor of which terminal enzyme was primarily used since a kinetic characterization of these enzymes is not available. Regardless, the thermodynamic modeling predicts that, under all conditions, H_2_ would be produced during lactate elongation (Table 2) and that TE could be a better terminal enzyme to force production of longer-chain acids in order to maximize ATP yield (Table 2).

In modeling scenarios utilizing lactate plus carboxylic acids as growth substrates (Table 2, equations 25 to 36), their elongation mediated by EUB1 would increase the amount of ATP it could produce compared to the use of lactate as a sole substrate only if using a terminal CoAT (Table 2, equations 19 to 21). H_2_ production or consumption is not predicted in these scenarios, and the calculated free energy released per mole of ATP produced (−50 to −53 kJ mol^−1^ ATP) is low, near the physiological limit of −60 kJ mol^−1^ ATP for energy conservation by the cell. Models with TE as the terminal enzyme in reverse β-oxidation were also analyzed (Table 2, equations 31 to 36) even though EUB1 is not predicted to have this ability as it lacks the ACS and ACK needed to utilize acetate (Fig. 4B). In such models, producing C6 and C8 from lactate plus acetate (Table 2, equations 32 to 33) is energetically favorable, whereas C4 production (Table 2, equation 31) is not.

## DISCUSSION

**FIG 5:**
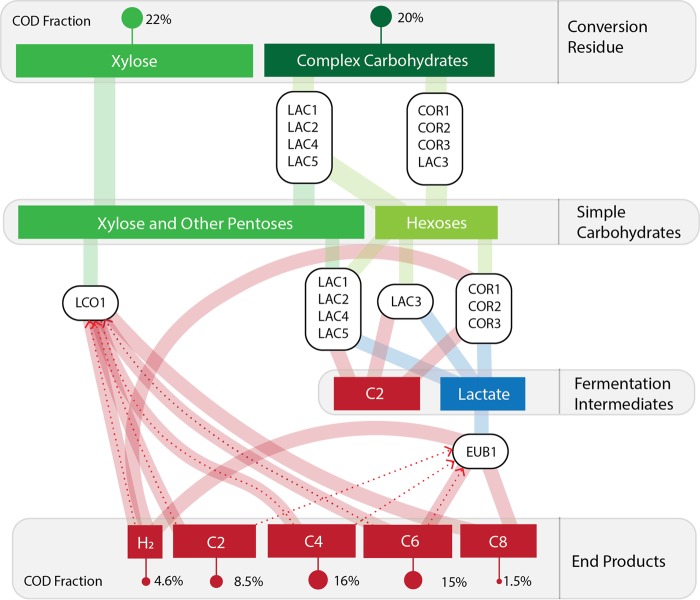
Predicted transformations of major substrates in conversion residues to MCFA by this anaerobic microbiome. The microbes in the LAC and COR bins are predicted to produce sugars from complex carbohydrates. Simple carbohydrates, including xylose remaining in conversion residues, are converted to lactate and acetate (C2) by *Lactobacillus* (LAC) and *Coriobacteriaceae* (COR) MAGs. The *Lachnospiraceae* (LCO1) MAG converts pentoses directly to butyric acid (C4). The *Eubacteriaceae* (EUB1) MAG produces hexanoic acid (C6) and octanoic acid (C8) from lactate. Further, LCO1 may utilize hydrogen to elongate C2 and C4 to MCFA, as represented by dashed lines. Additionally, EUB1 may elongate C2, C4, and C6 to C8.

In this report, we combined genomic, computational, and thermodynamic predictions to elucidate how a microbial community can convert organic substrates in lignocellulosic conversion residues into MCFA (Fig. 5). Specifically, this approach predicts that the coordinated and stepwise metabolic activity of different members of this microbiome allows cleavage of complex five- and six-carbon containing polysaccharides; conversion of sugars into simple fermentation products; and utilization of sugars and intermediate fermentation products for MFCA production. This approach further predicts the role of intracellular and extracellular reductants in these processes. Below, we illustrate the new insight that has been gained into the activity of a MCFA-producing microbiome and how this might relate to other systems.

The microbial community studied here is similar in phylogenetic composition to other microbial communities producing MCFA. For instance, in a community fed with dilute ethanol and stillage, *Lactobacillus* and a member of *Clostridium* group IV were abundant (12). In a system producing MCFA using thin stillage produced from corn ethanol, *Lactobacillus* levels were enriched alongside those of *Megasphaera*, a known MCFA-producing *Firmicute* (10). In a reactor converting waste from wine production to MCFA, levels of *Lactobacillus* and *Clostridia* related to *Ruminococcus* were enriched (9). In each case, a community containing carbohydrate-fermenting organisms and potential MCFA-producing organisms emerged.

Our data suggest that the contribution of *Lactobacillus* in this microbiome is in extracellular carbohydrate degradation and subsequent metabolism of pentose- and hexose-containing carbohydrates, while *Coriobacteriaceae* are predicted to metabolize hexose-containing carbohydrates. The combined metabolic activities of these two MAGs would produce oligosaccharides and monomeric sugars that would become available to these and other members of the microbiome. Metabolic reconstruction combined with microbiome transcript levels also suggested that the *Lactobacillus* and *Coriobacteriaceae* MAGs produce fermentation end products, primarily lactate and acetate, from these carbohydrates. *Coriobacteriaceae* spp., however, are also predicted to produce H_2_. In addition, microbiome gene expression patterns indicate that two MAGs, EUB1 and LCO1, produce MCFA via reverse β-oxidation. LCO1 is predicted to consume xylose on the basis of gene expression analysis, whereas RNA abundance measurements indicate that EUB1 consumes lactate.

We used thermodynamics to analyze hypothetical scenarios of MCFA production by EUB1 and LCO1. Although the comparison of these hypothetical scenarios did not provide an unequivocal answer regarding how chain elongation occurs in LCO1 and EUB1, it is helpful to generate hypotheses that could eventually be tested in future research. Our thermodynamic analysis suggests that the most energetically advantageous metabolism for LCO1 (on the basis of ATP production per mole of xylose consumed) is the consumption of xylose, H_2_, and carboxylates to produce C4, C6, and C8 while utilizing CoAT as a terminal enzyme. While xylose is a major component of conversion residue (CR; Fig. 1), H_2_ is expected to be produced by *Coriobacteriaceae* MAGs and EUB1. For EUB1, which is expected to utilize lactate, our analysis suggests that production of MCFA produces higher amounts of ATP, with production of C6 resulting in a 2-fold increase in ATP production versus production of C4 when lactate is consumed as a sole substrate.

Predictions from our thermodynamic modeling indicate that C8 production from lactate is energetically advantageous. However, this is at odds with C6 being produced from conversion residue at higher concentrations than C8 (Fig. 1). It is known that C8 is a biocide, so it may be that C8 accumulation is limited by the level of tolerance that community members have for this product (12). It is also possible that higher C6 production indicates a more important role of C6 production by LCO1 without lactate being an intermediate metabolite. It has also been shown that removal of C8 allows higher productivities of carboxylate platform systems (1).

H_2_ production and interspecies H_2_ transfer are known to have significant impacts on the metabolism of microbial communities (35). Our analysis predicts a role of H_2_ in supporting chain elongation in a carboxylate platform microbiome. While high H_2_ partial pressures are proposed to inhibit production of acetate and other carboxylic acids (36, 37), organisms that use the phosphoketolase pathway (the *Lactobacillus* and LCO1 MAGs identified in this study) can produce acetate, C4, and C8 without producing H_2_ (Table 1, equations 2, 3 and 5). While conversion of lactate to MCFA (Table 2, equations 19 to 24) is predicted to produce H_2_, other processes such as coutilization of xylose and a monocarboxylic acid for MCFA production (Table 1, equations 9 to 18) would result in consumption of H_2_. Therefore, H_2_ accumulation is not expected to limit production of MCFA, although H_2_ partial pressures may influence the metabolic routes utilized by the microbiome.

In considering how to further improve the production of MCFA with a microbiome, additional work is needed to characterize and engineer reverse β-oxidation proteins from the *Firmicutes* in order to improve production of organic acids longer than C4. Further, our data predict that the terminal enzyme of reverse β-oxidation can influence production of MCFA. While the presence of a CoAT enzyme results in higher ATP production, that of a TE makes production of MCFA more energetically advantageous by increasing the ATP yield for production of C6 and C8 compared to C4 (Tables 1–2). Therefore, engineering chain-elongating organisms to have only a TE rather than a CoAT may improve production of MCFA.

Our metabolic reconstructions suggest that lactate was a key fermentation product that supported MCFA production. Therefore, strategies to enhance lactate production and minimize the levels of other fermentation products (fermentation of carbohydrates to lactate rather than acetate in this example), could improve production of desired end products. Moreover, designing strategies to enrich a community that produces a critical intermediate such as lactate by one pathway (e.g., a community of homofermentative lactate-producing *Lactobacilli* rather than heterofermenters producing both lactate and acetate) could improve the performance of the microbiome. However, the principles relating to controlling the presence or dominance of heterofermentative versus homofermentative organisms in microbial communities remain largely unexplored. Alternatively, higher production of a desired product, C8, could be achieved by adjusting the abundance or by establishing a defined coculture containing a lactic acid bacterium capable of complex carbohydrate degradation, such as LAC1, and a lactate-elongating organism, such as EUB1. The ability to establish defined synthetic communities, to adjust the abundance of microbiome members, or to regulate the metabolic routes within the microbiome might allow more control over the function of a microbiome for production of MCFA or optimization of other traits.

In summary, this work demonstrates that one can dissect and model the composition of microbiomes as a way to understand the contribution of different community members to its function. In the case of an anaerobic carboxylate platform microbiome fed lignocellulosic ethanol conversion residue, two *Clostridia*-related organisms (EUB1 and LCO1) are predicted to be responsible for production of MCFA via reverse β-oxidation. This provides a genome-centered rationale for the previously established correlation between *Clostridia*-related abundance and MCFA production noted in carboxylate platform systems (4, 12). The results of this study further suggest that the terminal enzyme in product synthesis and the fermentation end products produced by other community members can play a role in determining the predominant products of this microbiome. These approaches, concepts, and insights should be useful in predicting and controlling MCFA production by reactor microbiomes and in analyzing the metabolic, genomic, and thermodynamic factors influencing the function of other microbiomes of health-related, environmental, agronomic, or biotechnological importance.

## MATERIALS AND METHODS

### Production of conversion residue.

Switchgrass used to generate conversion residue was treated by ammonia fiber expansion and enzymatically treated with Cellic CTec3 and Cellic HTec3 (Novozymes) to digest cellulose and hemicellulose (to produce glucose and xylose, primarily) (38). Hydrolysate was fermented with Saccharomyces cerevisiae Y128, a strain with improved xylose utilization (39). Fermentation media were distilled to remove ethanol (4).

### Bioreactor operation.

The bioreactor was seeded with acid digester sludge from the Nine Springs Wastewater Treatment Plant in Madison, WI. The retention time of the semicontinuous reactor was maintained at 6 days by pumping conversion residue into the reactor, pumping reactor effluent from the reactor once per hour, and maintaining a liquid volume of 150 ml in the reactor. The reactor contents were mixed by the use of a magnetic stir bar. The temperature of the reactor was controlled at 35°C using a water bath, and the pH of the reactor was maintained at 5.5 by feeding 5 M KOH through a pump connected to a pH controller. This reactor sustained MCFA production for 252 days (4).

### Metabolite analysis.

Samples from the bioreactor and conversion residue were collected for metabolite analysis. All samples were filtered using 0.22-μm-pore-size syringe filters (SLGP033RS; ThermoFisher Scientific, Waltham, MA, USA). Chemical oxygen demand (COD) analysis was performed using high-range COD digestion vials (2125915; Hach, Loveland, CO, USA) per standard methods (40). Soluble carbohydrates were measured with the anthrone method (41). Glucose, xylose, acetic acid, formic acid, lactic acid, succinic acid, pyruvic acid, glycerol, and xylitol were analyzed with high-performance liquid chromatography and quantified with an Agilent 1260 Infinity refractive index detector (Agilent Technologies, Inc. Palo Alto, CA) using a 300-by-7.8-mm Bio-Rad Aminex HPX-87H column with a Cation-H guard (Bio-Rad, Inc., Hercules, CA). Acetamide, ethanol, n-propionic acid, n-butyric acid, iso-butyric acid, n-pentanoic acid, iso-pentanoic acid, n-hexanoic acid, iso-hexanoic acid, n-heptanoic acid, and n-octanoic acid were analyzed with tandem gas chromatography-mass spectrometry (GC-MS). An Agilent 7890A GC system (Agilent Technologies, Inc. Palo Alto, CA) with a 0.25 mm Restek Stabilwax DA 30 column (model 11008; Restek, Belefonte, PA) was used. The GC-MS system was equipped with a Gerstel MPS2 auto sampler (Gerstel, Inc., Baltimore, MD) and a solid-phase microextraction gray hub fiber assembly (Supelco, Bellefonte, PA). The MS detector was a Pegasus 4D time of flight mass spectrometer (TOF-MS) (Leco Corp., Saint Joseph, MI). Stable isotope-labeled internal standards were used for each of the analytes measured with GC-MS.

### DNA and RNA sequencing.

Biomass samples, consisting of centrifuged and decanted 2-ml aliquots, were collected at day 12, day 48, day 84, day 96, and day 120 of reactor operations from the day of initial startup. Samples were also taken at 96 days and flash-frozen in liquid nitrogen for RNA extraction. For DNA extraction, cells were lysed by incubation in a mixture of a lysis solution (1.5 M sodium chloride, 100 mM Tris, 100 mM ethylenediamine [EDTA], 75 mM sodium phosphate, 1% cetyltrimethylammonium bromide, 2% sodium dodecyl sulfate [SDS]), lysozyme (Thermo Fisher Scientific, MA, USA), and proteinase K (New England Biolabs, MA, USA). We then added 500 μl of a 24:24:1 solution of phenol, chloroform, and isoamyl alcohol and subjected samples to bead beating for 2 min. After the bead beating was completed, biomass was centrifuged at 5,000 relative centrifugal force (rcf) for 3 min and the entire supernatant was transferred to a 1.5-ml centrifuge tube. Samples were centrifuged again at 12,000 rcf for 10 min, and the aqueous layer was then removed to a new centrifuge tube. A second phase separation procedure was then performed using chloroform. After centrifuging again and separating the aqueous phase, 500 μl of isopropanol was added to each sample and the samples were then incubated at -20°C for 24 h. Following this incubation, samples were centrifuged at 12,000 rcf for 30 min at 4°C, decanted, and washed with 70% ethanol. After air-drying of the samples, the pellets were resuspended in 100 μl of Tris-EDTA buffer and 2 μl of 10 mg/ml RNase was added to each sample. Samples were incubated for 15 min at 37°C. We then added 100 μl of a 24:24:1 solution of phenol, chloroform, and isoamyl alcohol to each sample and centrifuged the reaction mixture at 12,000 rcf for 10 min. We separated the aqueous phase to a new centrifuge tube and added 100 μl of chloroform. Again, samples were centrifuged at 12,000 rcf for 10 min and the aqueous phase was separated to a new centrifuge tube. We then added 10 μl of 3 M sodium acetate and 250 μl of 95% ethanol to each sample and incubated for 24 h at −20 °C. Samples were centrifuged at 12,000 rcf for 30 min at 4 °C, and the pellets were washed with 70% ethanol. After air-drying, pellets were resuspended in 50 μl of Tris-EDTA buffer. After resuspension of the DNA, quantity, purity, and quality were assessed with a Qubit 4 Fluorometer (Thermo Fisher Scientific, MA, USA), a Nanodrop 2000 spectrophotometer (Thermo Fisher Scientific, MA, USA), and gel electrophoresis.

For RNA extraction, cells were lysed by incubation in a lysis solution (20 mM sodium acetate, 1 mM EDTA, and 0.5% SDS prepared in water treated with diethylpyrocarbonate [Invitrogen, CA, USA]) and TRIzol (Invitrogen, CA, USA). The treated cells were subjected to 2 min of bead beating using lysing matrix A (MP Biomedicals, CA, USA). After this step, successive phase separation steps performed with mixtures of phenol, chloroform, isoamyl alcohol, and chloroform were used to separate nucleic acids from additional cell material, as described above. RNA was further purified with an RNEasy minikit (Qiagen, Hilden, Germany) and on-column DNase 1 (Qiagen, Hilden, Germany) treatment. After resuspension of the RNA, quantity, purity, and quality were assessed with a Qubit 4 fluorometer (Thermo Fisher Scientific, MA, USA), a Nanodrop 2000 spectrophotometer (Thermo Fisher Scientific, MA, USA), and gel electrophoresis. RNA samples were submitted to the University of Wisconsin Gene Expression Center for quality control with a Bioanalyzer (Agilent, CA, USA), and rRNA reduction was performed with a RiboZero-Bacteria rRNA removal kit (Illumina, CA, USA) with a 1-μg RNA input. Strand-specific cDNA libraries were prepared with a TruSeq RNA library preparation kit (Illumina, CA, USA).

DNA and RNA were sequenced with an Illumina HiSeq 2500 platform (Illumina, CA, USA). For DNA, an average insertion size of 550 bp was used and 2 × 250-bp reads were generated. For RNA, 1 × 100-bp reads were generated. Raw DNA and cDNA read data can be found on the National Center for Biotechnology Information (NCBI) website (see below).

### Metagenomic assembly, binning, and quality control.

DNA sequencing reads were filtered using Sickle with a minimum quality score of 20 and a minimum sequence length of 100 (42). Reads from all five samples were then coassembled using metaspades and kmer values of 21, 33, 55, 77, 99, and 127 (43). Binning of assembled contigs was performed with MaxBin v2.2.1 (44) (see Table S1 in the supplemental material). The quality, completeness, and contamination of each bin were analyzed with CheckM v1.0.3 (45). Read mapping was performed with BBMAP v35.92 (https://sourceforge.net/projects/bbmap) to estimate the relative abundance of each bin. Relative abundance was calculated by normalizing the number of mapped reads to the genome size.

10.1128/mSystems.00221-18.3 TABLE S1 Summary of MAGs obtained from DNA sequence analysis of the reactor microbiome. Taxonomy, completeness, and contamination were estimated with CheckM. Draft genomes were assembled from five independent reactor samples. These MAGs represent the 10 most abundant MAGs at day 96 (Fig. 1). Download Table S1, DOCX file, 0.02 MB. Copyright © 2018 Scarborough et al. 2018 Scarborough et al. This content is distributed under the terms of the Creative Commons Attribution 4.0 International license.

### Phylogenetic analysis.

Phylogeny of the draft genomes was assessed using 37 universal single-copy marker genes with Phylosift v1.0.1 (46). In addition to the draft genomes, 62 publically available genomes of related organisms were used to construct a phylogenetic tree. Concatenated amino acid sequences of the marker genes were aligned with Phylosift, and a maximum likelihood phylogenetic tree was constructed with RAxML v8.2.4 with the PROTGAMMAAUTO model and 100 bootstraps (47). ANI calculations were performed using JSpecies (48).

### Genome annotations.

Draft genomes were annotated with MetaPathways v2.5 (23). Open reading frames (ORFs) were predicted using Prodigal v2.0 (49), and the ORFs were annotated with the following databases: SEED (accessed March 2013), Clusters of Orthologous Groups (COG; accessed December 2013), RefSeq (accessed January 2017), Metacyc (accessed October 2011), and KEGG (accessed January 2017). The LAST algorithim was used for assigning functional annotations (50). Functional annotations for each MAG are provided in Data Set S4 in the supplemental material. Draft genomes were further annotated using the CAZY database (24). CELLO was used to determine the subcellular location of the CAZYs (25). Transporters were identified using the Transporter Classification Database.

### Transcript analysis.

Analysis of transcript data was performed as described by Lawson et al. (51). cDNA reads were quality filtered as described above for DNA. SortMeRNA was used to remove rRNA sequences using multiple databases for RNA sequences (52). The remaining non-rRNA sequences were then mapped back to the draft genomes using BBMap v35.92 (https://sourceforge.net/projects/bbmap) with the minimum sequence identity set to 0.95. Ambiguous reads with multiple top-hit mapping locations were assigned to a random ORF. The number of RNA reads mapping to each ORF was calculated with htseq-count v0.6.1 with the “intersection-strict “parameter (53). Relative gene expression (quantified as reads per kilobase per million [RPKM]) was calculated for each ORF by normalizing the number of mapped RNA reads for each ORF to the ORF length and the total number of RNA reads mapping back to the genome. The relative number of RPKM (relPKM) was then calculated as the ratio of the RPKM value for the ORF to the median RPKM value across the draft genome. Finally, the log_2_(relRPKM) value was calculated to determine the log fold difference. As such, a positive number corresponds to greater-than-median expression levels and a negative number to expression below median levels.

### Data availability.

Raw DNA and cDNA read data can be found on the National Center for Biotechnology Information (NCBI) website under BioProject accession no. PRJNA418244. Sequencing reads are available through the following NCBI sequencing read archive (SRA) accession numbers: DNA day 12, SRR6292603; DNA day 48, SRR6292602; DNA day 84, SRR6292605; DNA day 96, SRR6292604; DNA day 120, SRR6292607; RNA day 96 A, SRR6292606; RNA day 96 B, SRR6292609; RNA day 96 C, SRR6292608.
