# Supplementary figures and images for: Metatranscriptomic and Thermodynamic Insights into Medium-Chain Fatty Acid Production Using an Anaerobic Microbiome
Source: mSystems. 2018 Nov 20;3(6):e00221-18. doi: 10.1128/mSystems.00221-18 (PMC6247018; doi:10.1128/mSystems.00221-18)

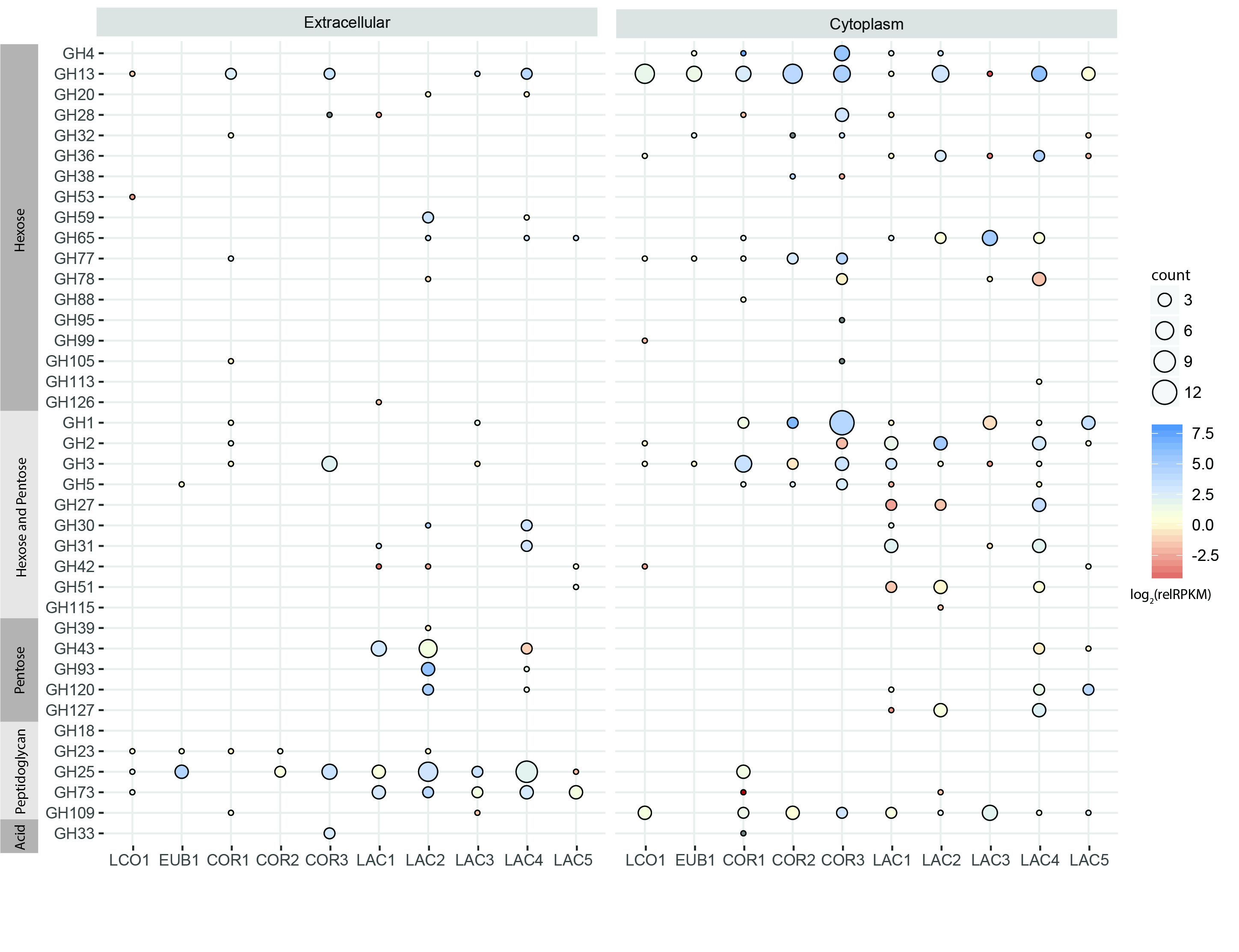

Supplement: FIG S1 [file sys006182291sf1.tif]

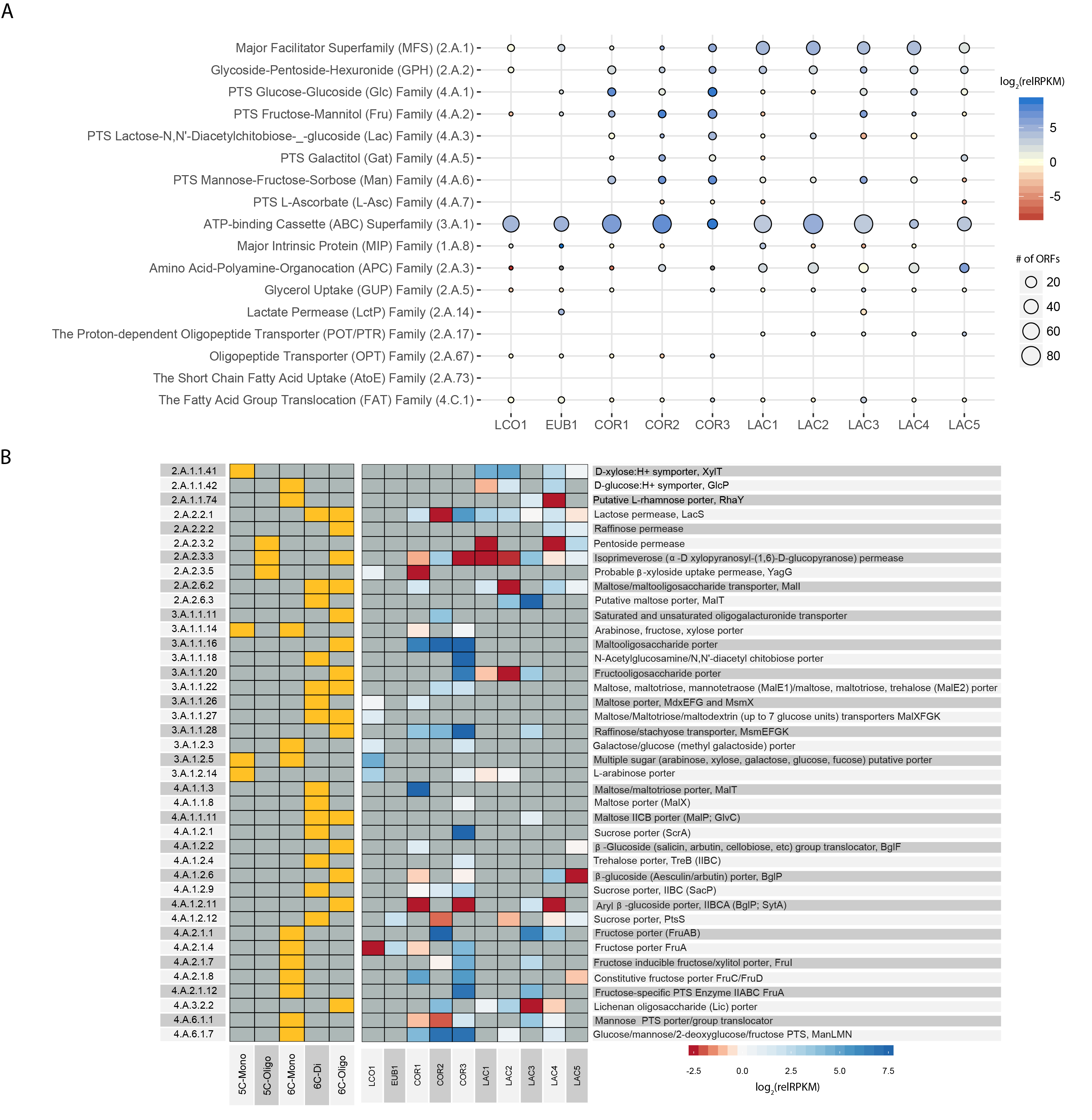

Supplement: FIG S2 [file sys006182291sf2.tif]
