# Supplementary material for: Metatranscriptomic and Thermodynamic Insights into Medium-Chain Fatty Acid Production Using an Anaerobic Microbiome
Source: mSystems. 2018 Nov 20;3(6):e00221-18. doi: 10.1128/mSystems.00221-18 (PMC6247018; doi:10.1128/mSystems.00221-18)
Supplement: DATA SET S7 [file sys006182291sd7.pdf]

Fiq S7.1 (Eq. 1)

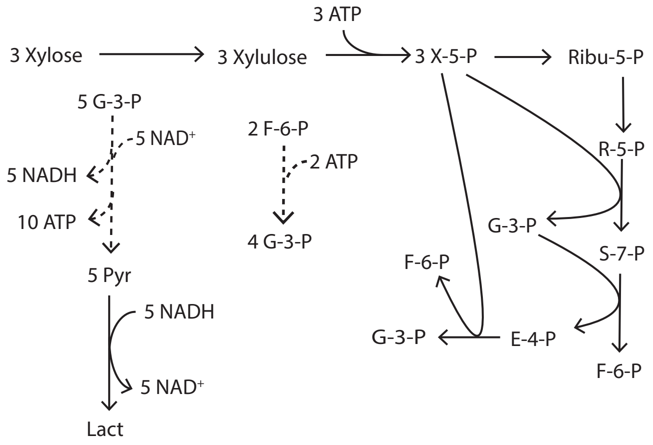

3 Xylose ⟶ 5 Lactate + 5 H^+ $\Delta G^{0\prime}$= -516kJ/reaction

ATP Yield: 1.67 ATP/ Xylose

Fiq S7.2 (Eq. 2)

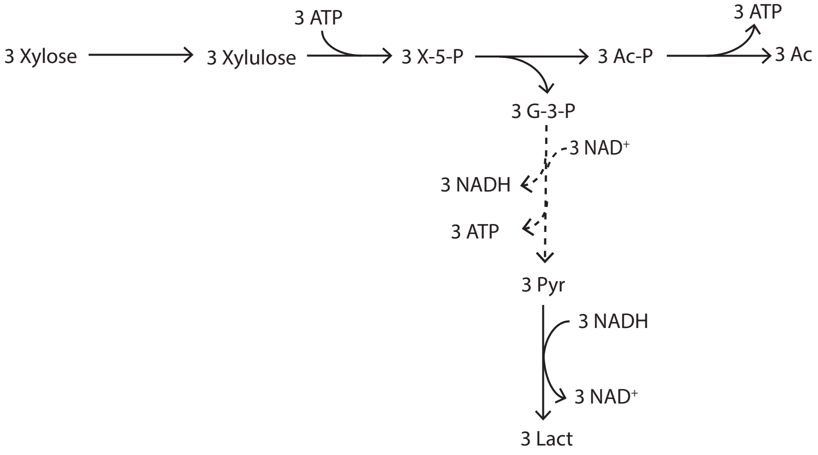

3 Xylose ⟶ 3 Lactate + 3 Acetate+ 6H^+ $\Delta G^{0\prime}$= -634kJ/reaction

ATP Yield: 2.00 ATP/ Xylose

Fiq S7.3 (Eq. 3)

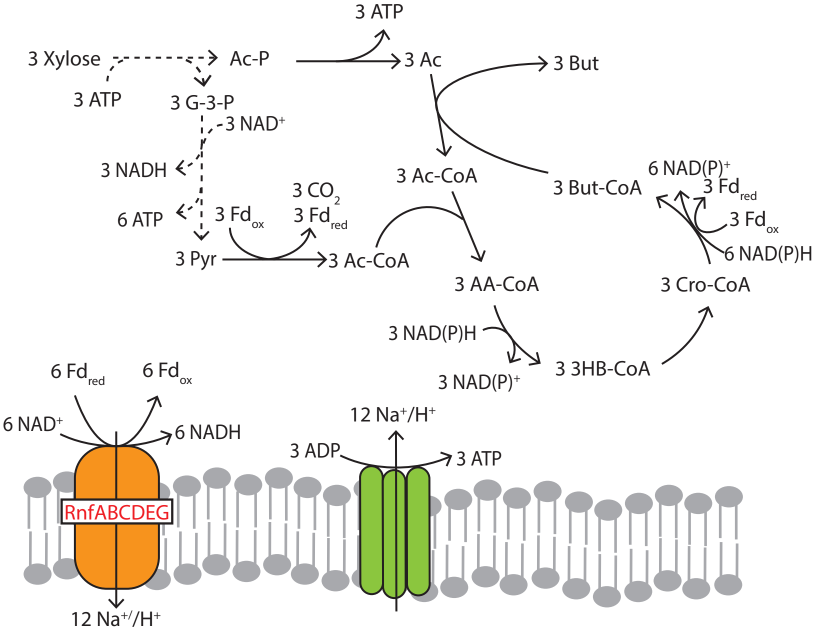

3 Xylose ⟶ 3 Butyrate + 3 H^++ 3 H_2O + 3 CO_2 $\Delta G^{0\prime}$= -787kJ/reaction

ATP Yield: 3.00 ATP/ Xylose

Fiq S7.4 (Eq. 4)

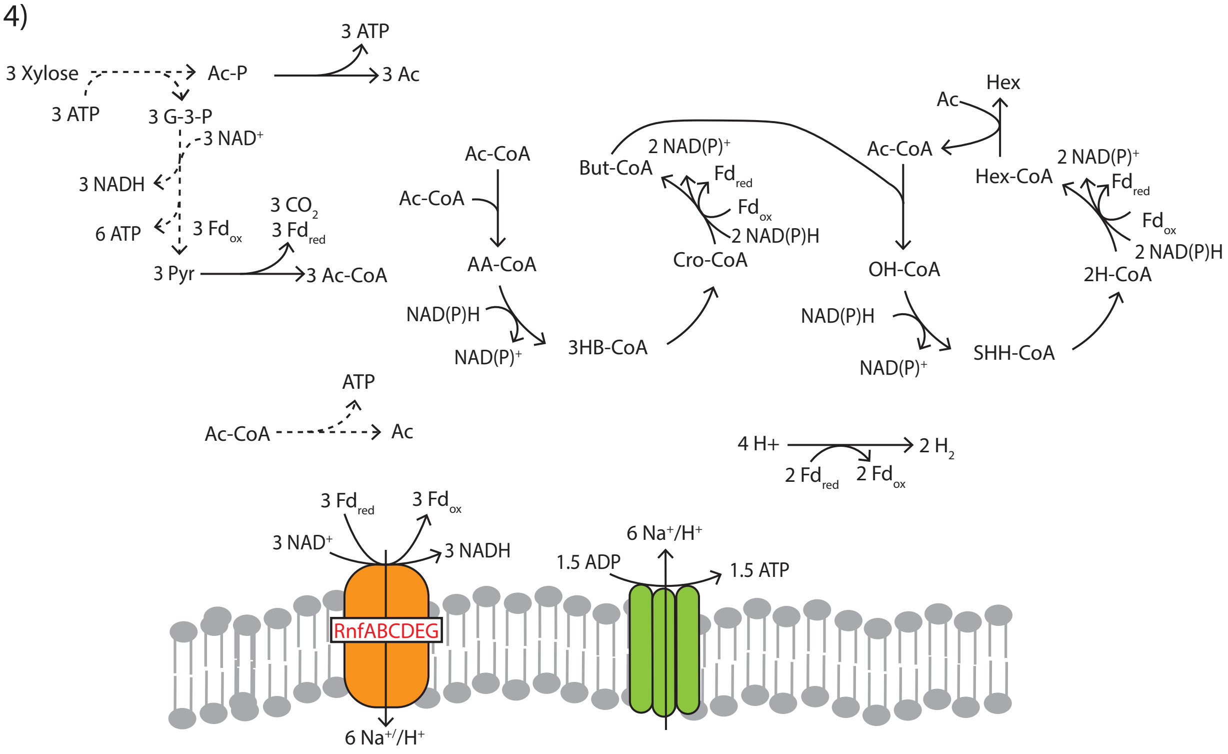

3 Xylose $\longrightarrow$ 1 Hexanoate + 3 Acetate+ 4 H^++ 1 H_2O + 3 CO_2 + 2 H_2 $\Delta G^{0\prime}$= -744 J/reaction

ATP Yield: 2.83 ATP/ Xylose

Fiq S7.5 (Eq. 5)

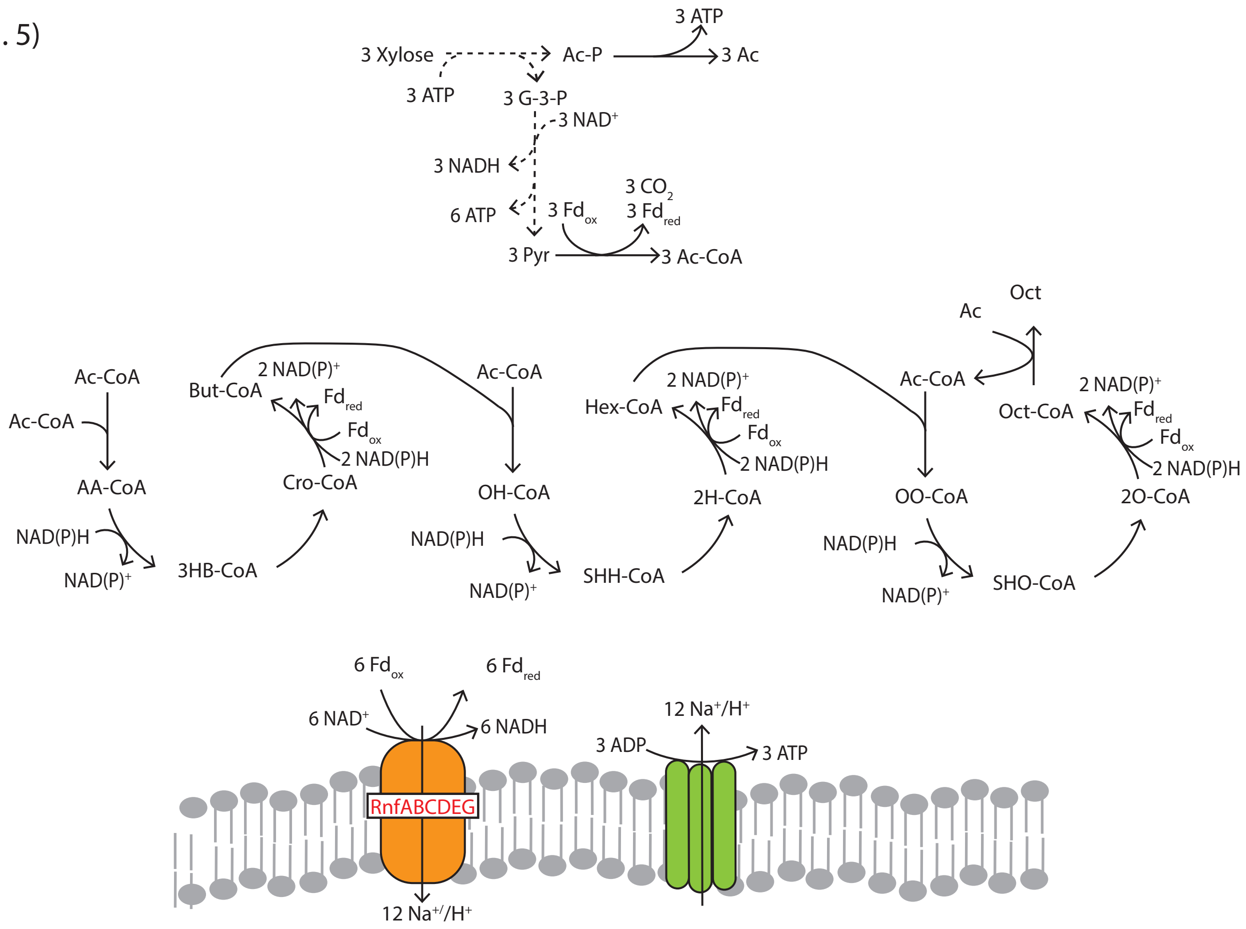

3 Xylose $\longrightarrow$ 1 Octanoate + 2 Acetate+ 3 H^++ 3 H_2O + 3 CO_2 $\Delta G^{0\prime}$= -790kJ/reaction

ATP Yield: 3.00 ATP/ Xylose

Fiq S7.6 (Eq. 6)

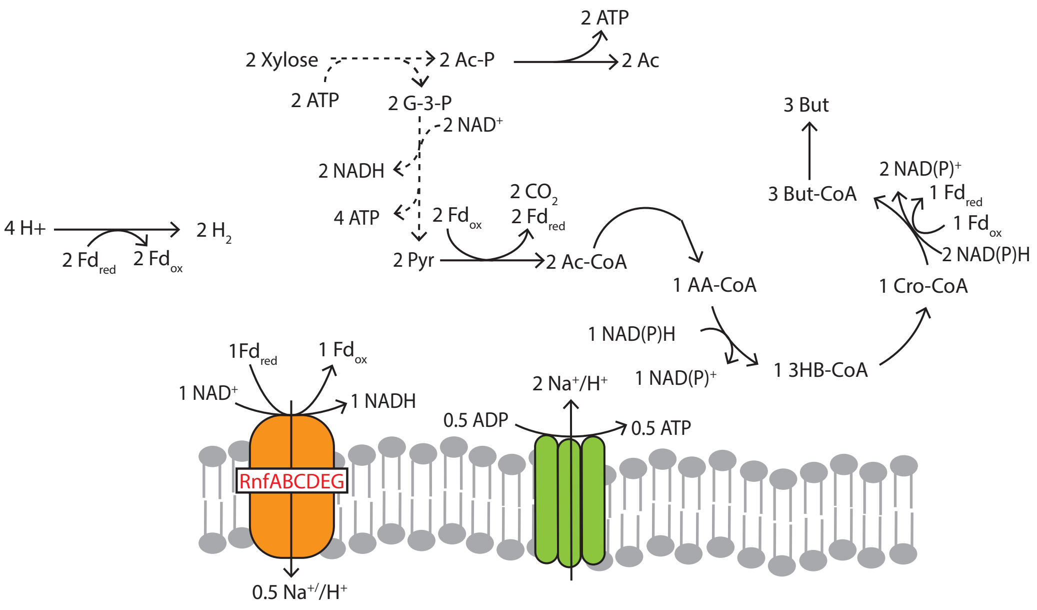

2 Xylose $\longrightarrow$ 2 Acetate + 1 Butyrate + $3\ H^{+}$ + $2\ CO_2$ + $2\ H_2$

ATP Yield: 2.25 ATP/ Xylose $\Delta G^{0'}$= -477 kJ/reaction

Fiq S7.7 (Eq. 7)

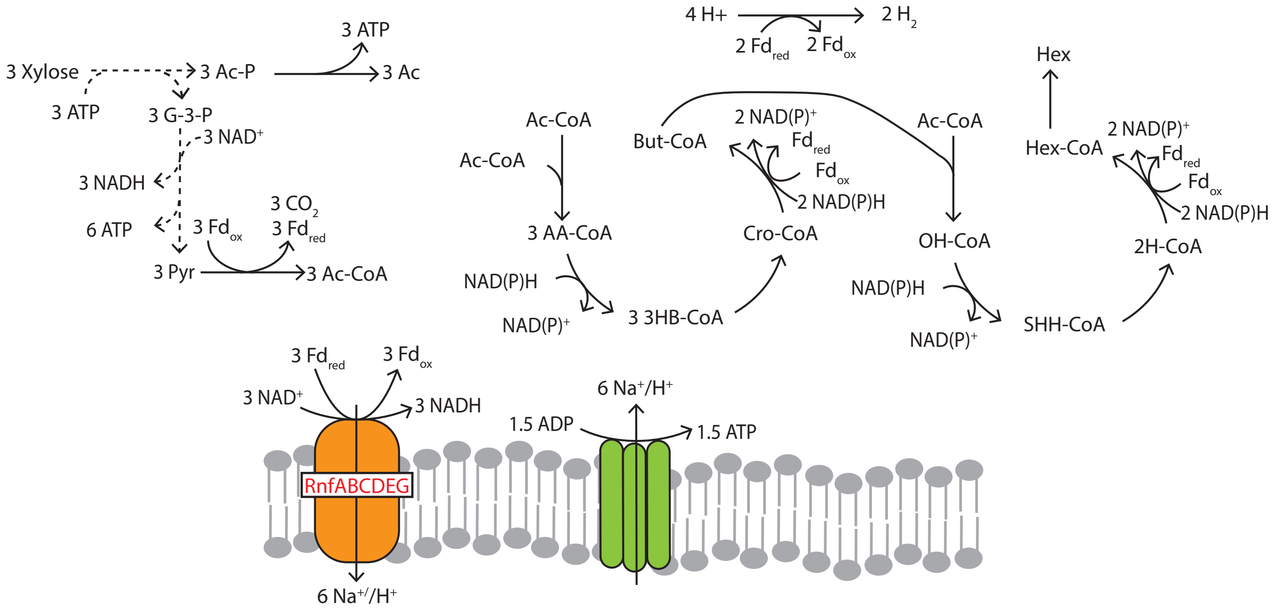

3 Xylose $\longrightarrow$ 3 Acetate + 1 Hexanaote + $4\ H^{+}$ + $3\ CO_2$ + $1\ H_2O$ + $2\ H_2$

ATP Yield: 2.50 ATP/ Xylose $\Delta G^{0'}$= -739 kJ/reaction

Fiq S7.8 (Eq. 8)

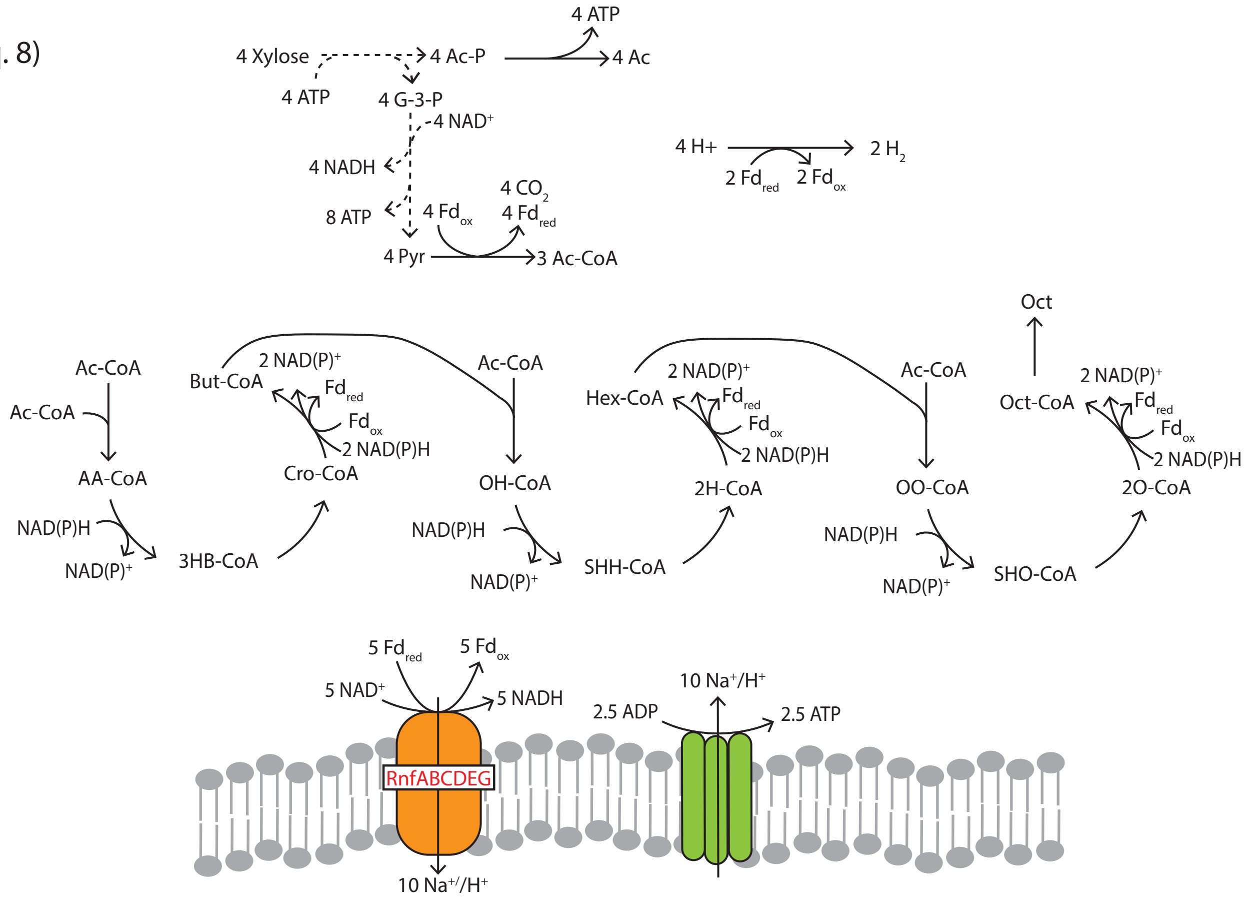

4 Xylose ⟶ 4 Acetate + 1 Octanoate + 5 H^+ + 4 CO_2 + 2 H_2O + 2 H_2

ATP Yield: 2.63 ATP/ Xylose $\Delta G^{0'}$= -1,005 kJ/reaction

Fiq S7.9 (Eq. 9)

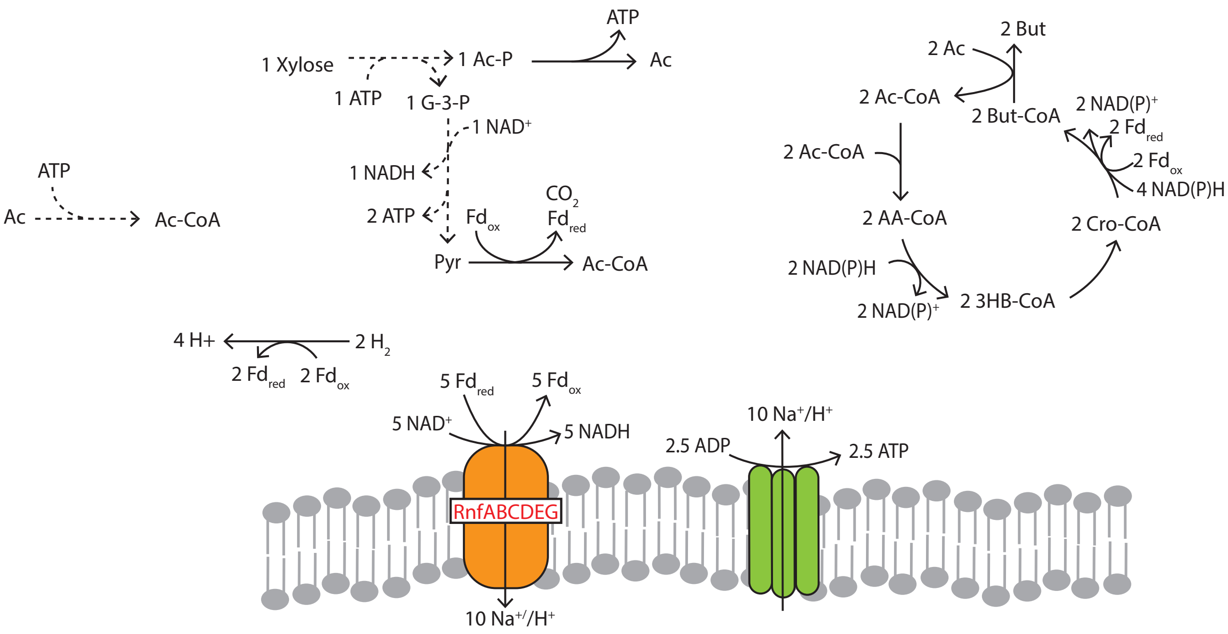

1 Xylose + 2 Acetate + 2 H_2 ⟶ 2 Butyrate + 1 CO_2 + 3 H_2O $\Delta G^{0'}$= -311kJ/reaction

ATP Yield: 3.50 ATP/ Xylose

Fiq S7.10 (Eq. 10)

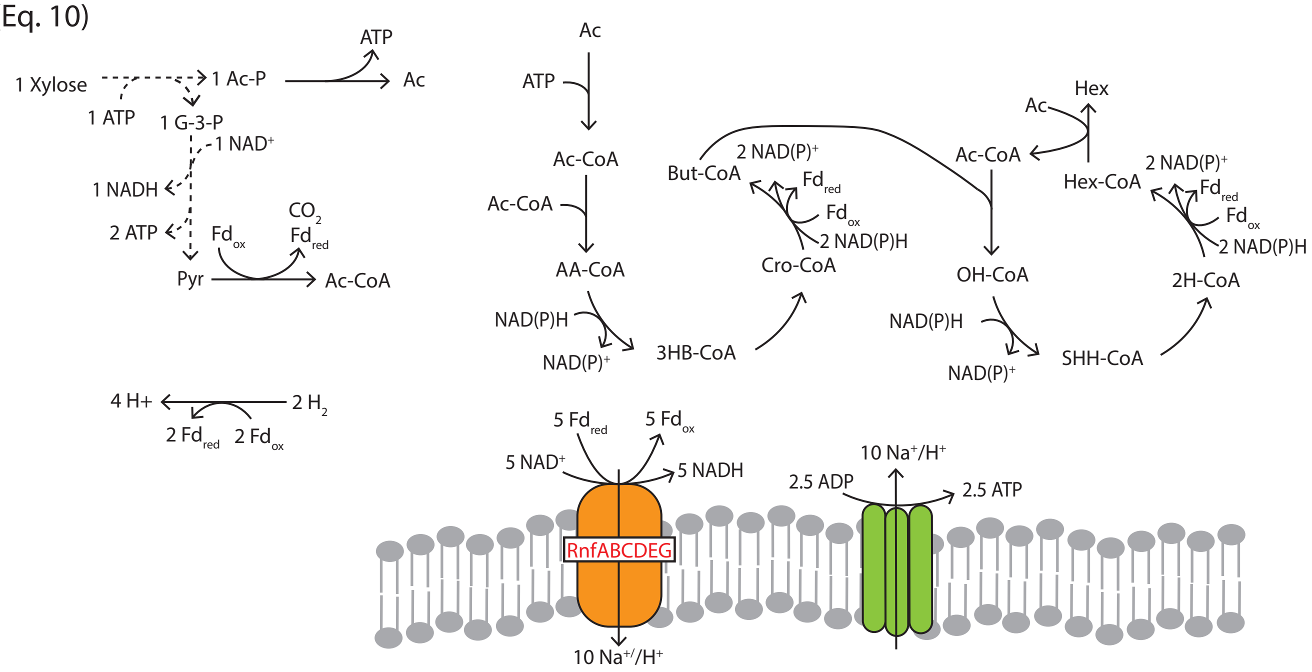

1 Xylose + 1 Acetate + 2 H_2 ⟶ 1 Hexanoate + 1 CO_2 + 3 H_2O $\Delta G^{0\prime}$= -311kJ/reaction

ATP Yield: 3.50 ATP/ Xylose

Fiq S7.11 (Eq. 11)

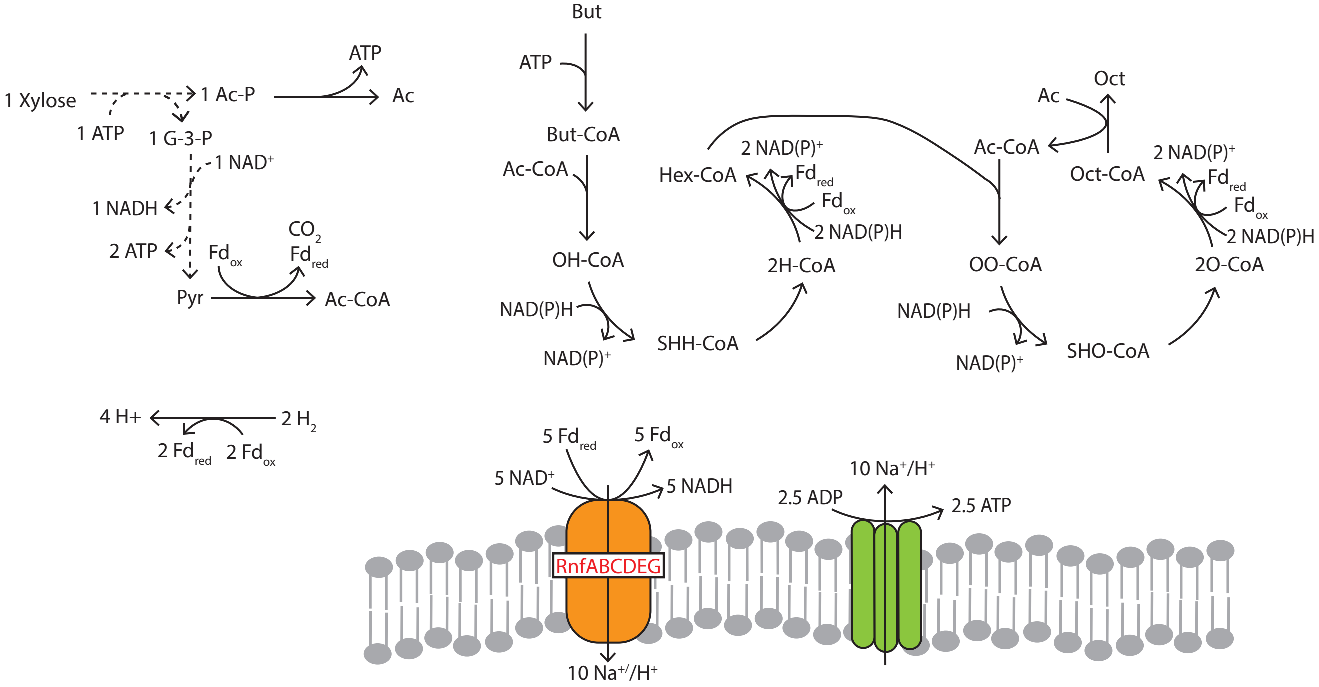

1 Xylose + 1 Butyrate + 4 H_2 ⟶ 1 Octanaote+ 1 CO_2 + 3 H_2O $\Delta G^{0\prime}$= -314kJ/reaction

ATP Yield: 3.50 ATP/ Xylose

Fiq S7.12 (Eq. 12)

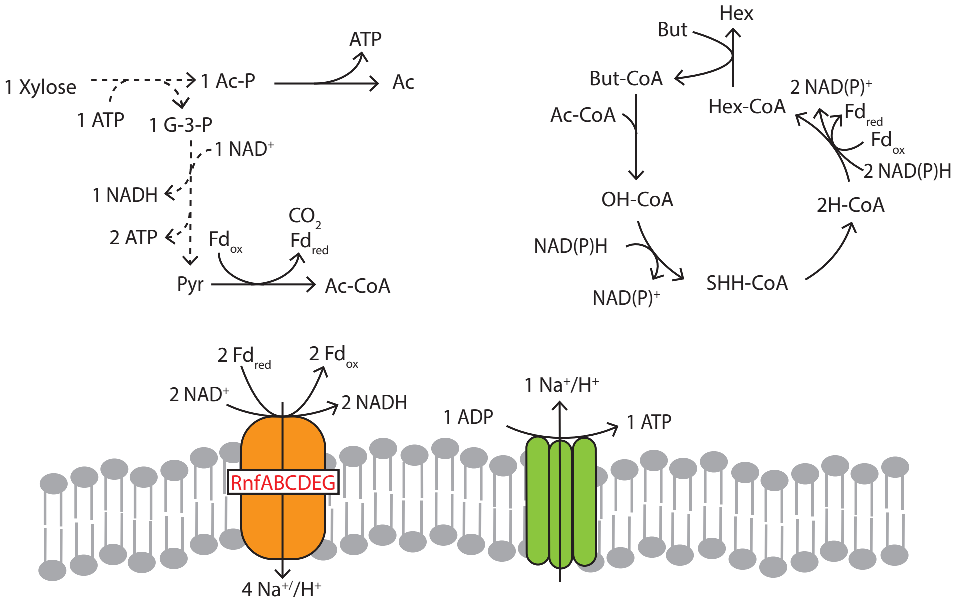

1 Xylose + 1 Butyrate ⟶ 1 Hexanaote + 1 Acetate + 1 CO_2 + 1 H_2O + 1 H^+ $\Delta G^{0'}$= -264kJ/reaction

ATP Yield: 3.00 ATP/ Xylose

Fiq S7.13 (Eq. 13)

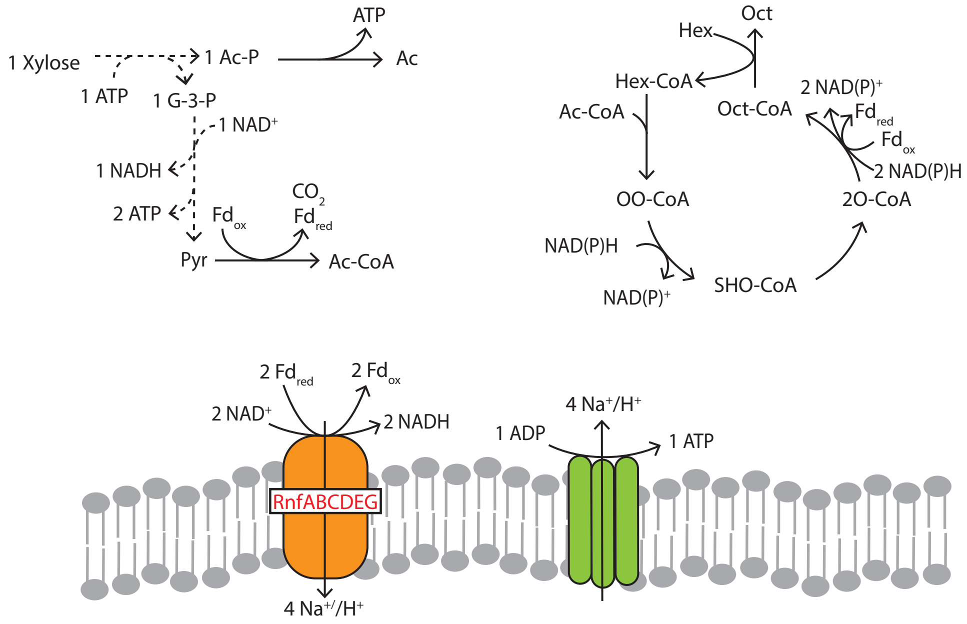

1 Xylose + 1 Hexanoate ⟶ 1 Octanaote + 1 Acetate + 1 CO_2 + 1 H_2O + 1 H^+ $\Delta G^{0'}$= -267kJ/reaction

ATP Yield: 3.00 ATP/ Xylose

Fiq S7.14 (Eq. 14)

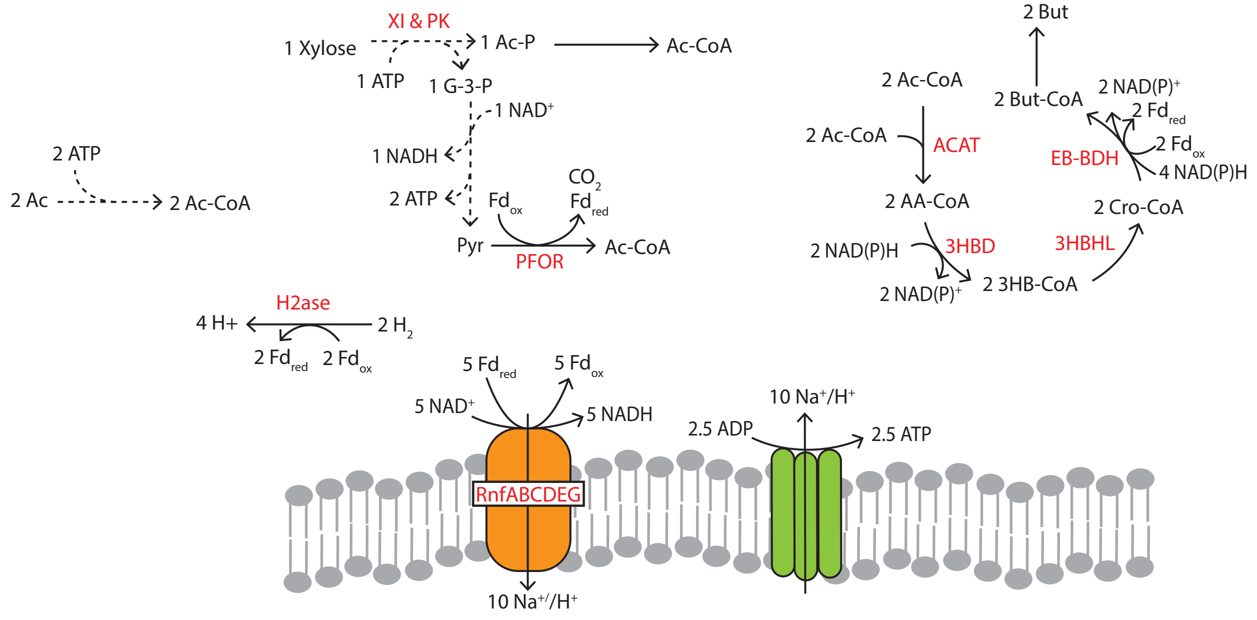

1 Xylose + 2 Acetate + 2 H_2 ⟶ 2 Butyrate + 1 CO_2 + 3 H_2O $\Delta G^{0'}$= -311kJ/reaction

ATP Yield: 1.50 ATP/ Xylose

Fiq S7.15 (Eq. 15)

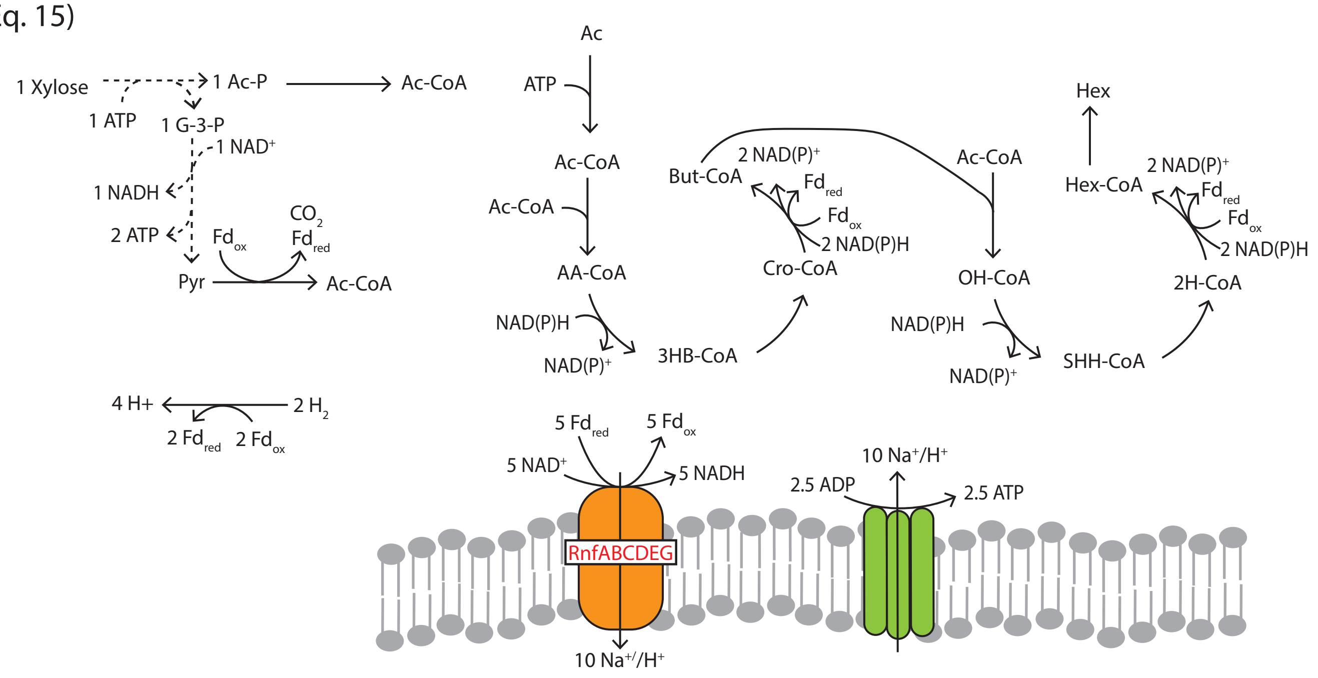

1 Xylose + 1 Acetate + 2 H_2 ⟶ 1 Hexanoate + 1 CO_2 + 3 H_2O $\Delta G^{0'}$= -311kJ/reaction

ATP Yield: 2.50 ATP/ Xylose

Fiq S7.16 (Eq. 16)

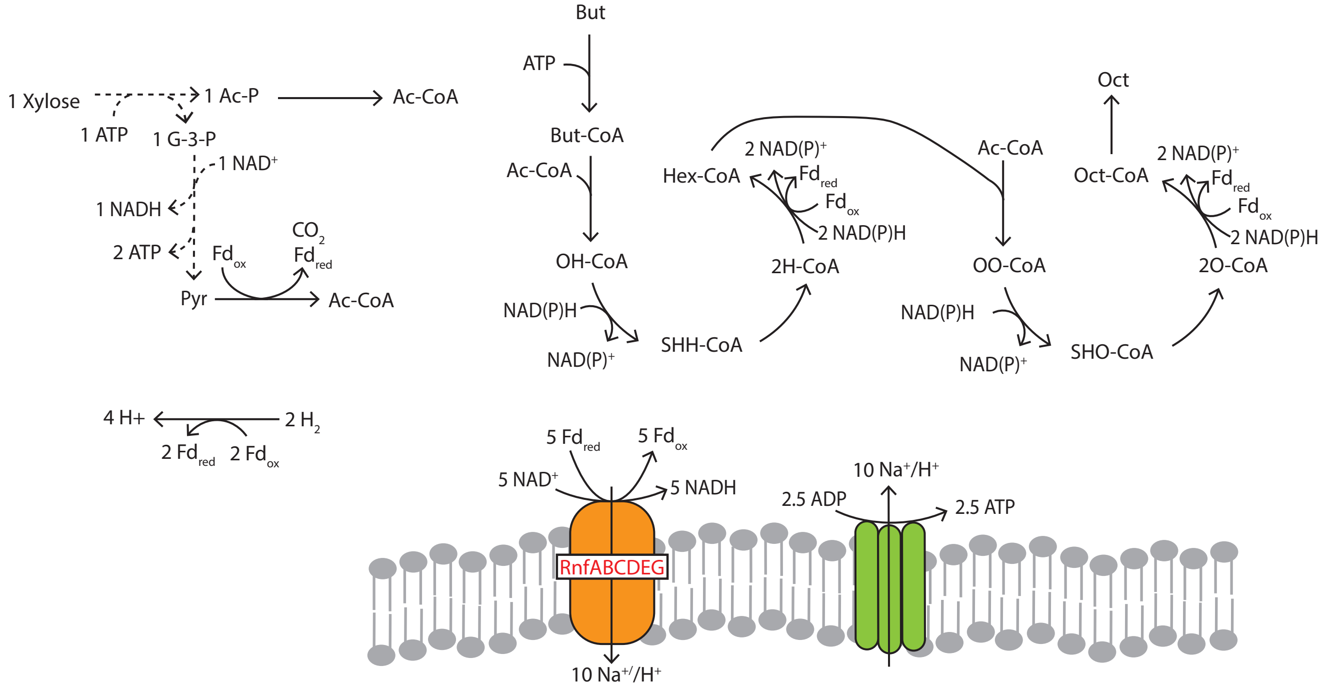

1 Xylose + 1 Butyrate + $4\ H_2 \longrightarrow 1$ Octanaote+ $1\ CO_2 + 3\ H_2O$ $\Delta G^{0'} = -314$kJ/reaction

ATP Yield: 2.50 ATP/ Xylose

Fiq S7.17 (Eq. 17)

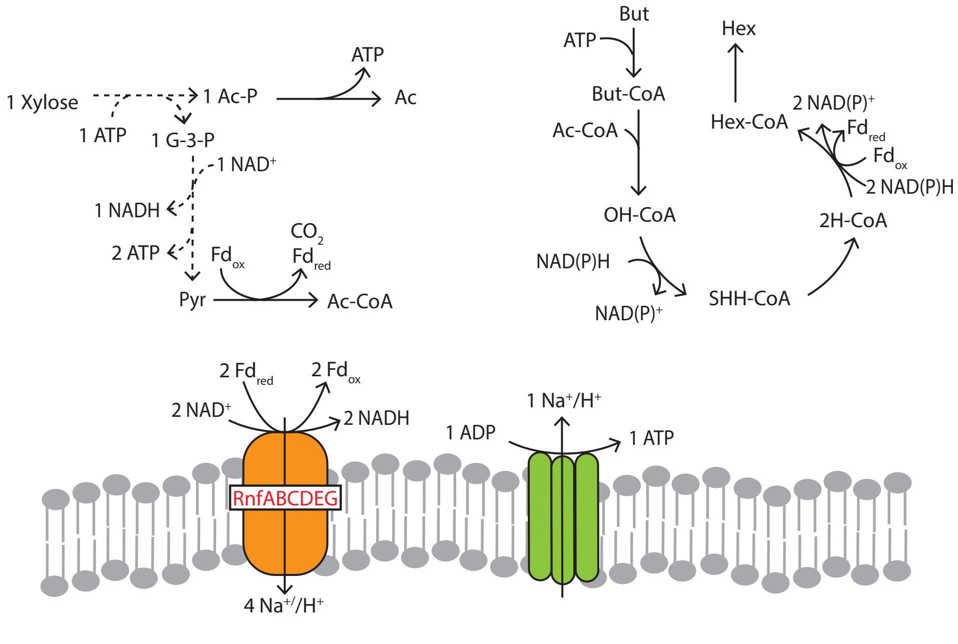

1 Xylose + 1 Butyrate $\longrightarrow 1$ Hexanaote + 1 Acetate + $1\ CO_2 + 1\ H_2O + 1\ H^+$ $\Delta G^{0'} = -264$kJ/reaction

ATP Yield: 2.00 ATP/ Xylose

Fiq S7.18 (Eq. 18)

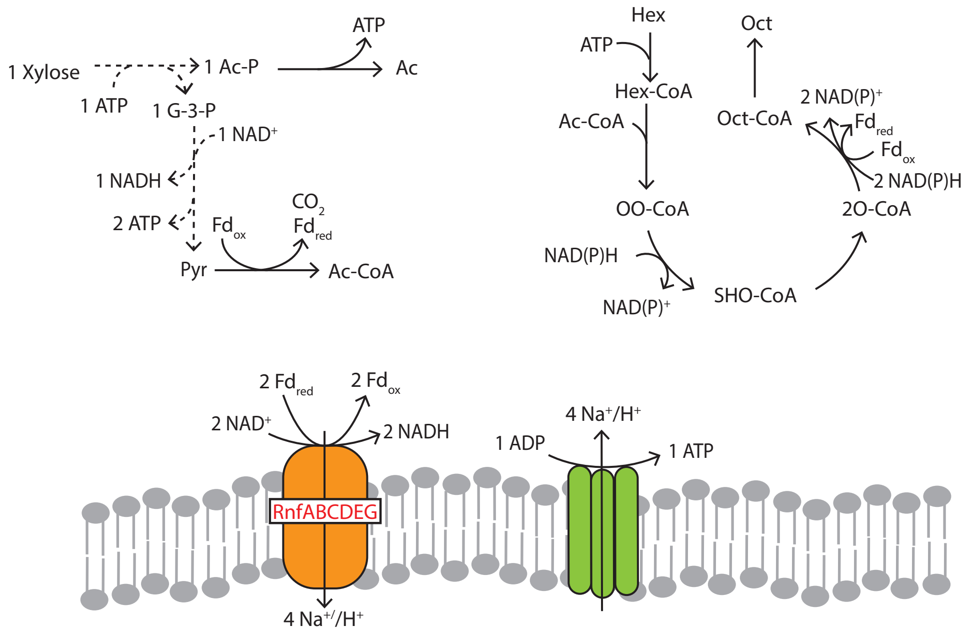

1 Xylose + 1 Hexanoate ⟶ 1 Octanaote + 1 Acetate + 1 CO_2 + 1 H_2O + 1 H^+ $\Delta G^{0'}$= -267kJ/reaction

ATP Yield: 2.00 ATP/ Xylose

Fiq S7.19 (Eq. 19)

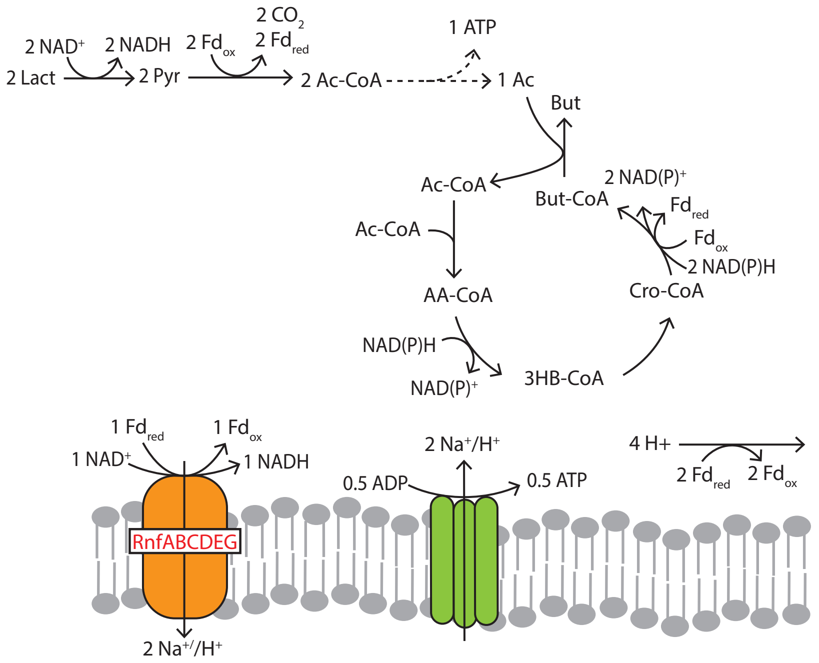

2 Lactate + 1 H^+ ⟶ 1 Butyrate + 2 H_2 + 2 CO_2 $\Delta G^{0'}$= -54.0kJ/reaction

ATP Yield: 0.75 ATP/ Lactate

Fiq S7.20 (Eq. 20)

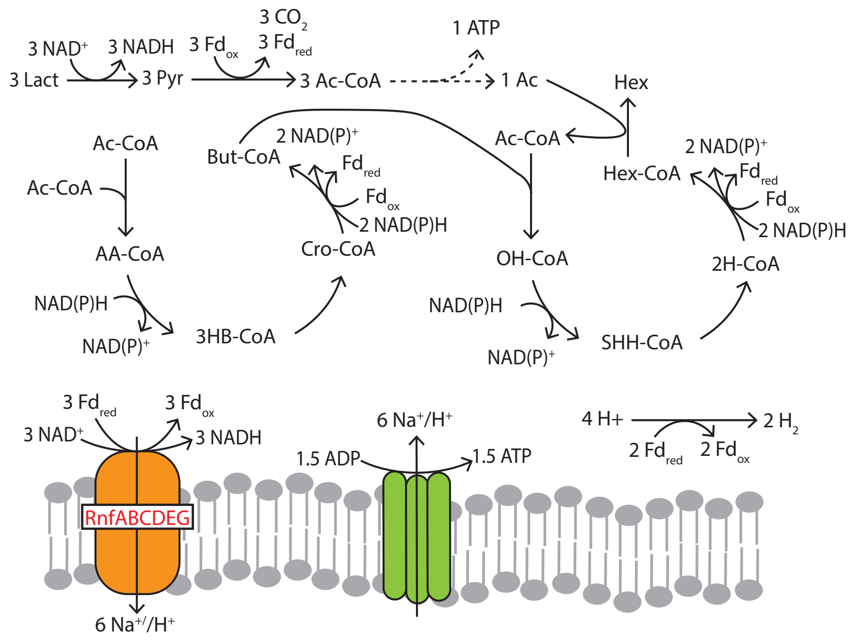

3 Lactate + $2\ H^+ \longrightarrow 1$ Hexanoate + $2\ H_2$ + H_2O + $3\ CO_2$ $\Delta G^{0\prime}$= -105kJ/reaction

ATP Yield: 0.83 ATP/ Lactate

Fiq S7.21 (Eq. 21)

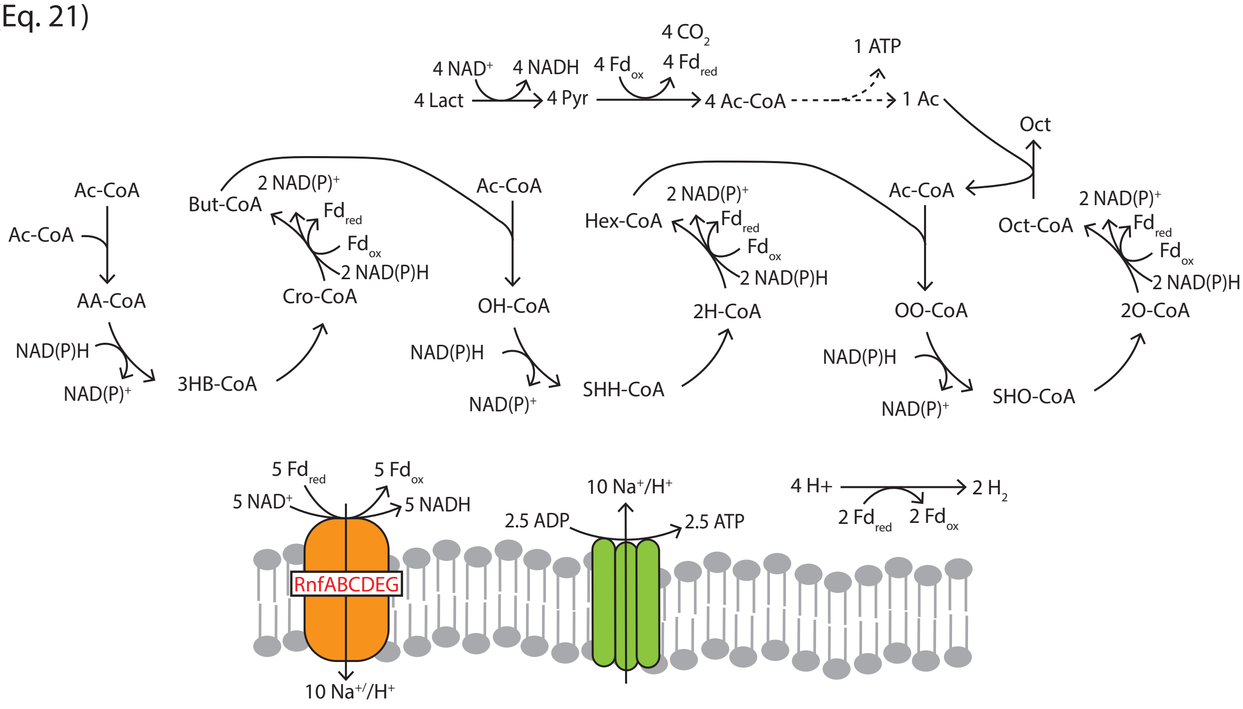

4 Lactate + $3\ H^+ \longrightarrow 1$ Octanoate + $2\ H_2$ + $2\ H_2O$ + $4\ CO_2$ $\Delta G^{0\prime}$= -156kJ/reaction

ATP Yield: 0.88 ATP/ Lactate

Fiq S7.22 (Eq. 22)

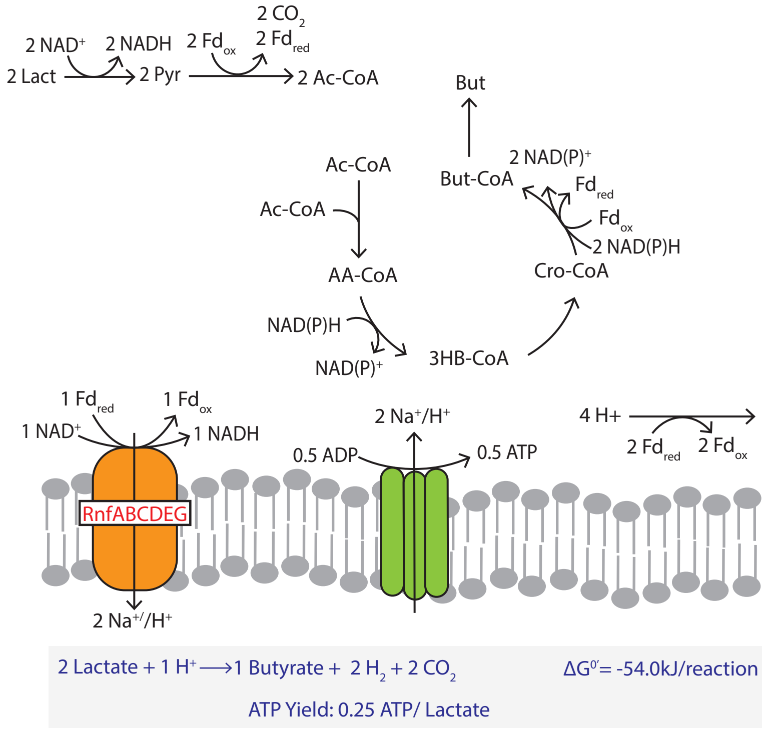

Fiq S7.23 (Eq. 23)

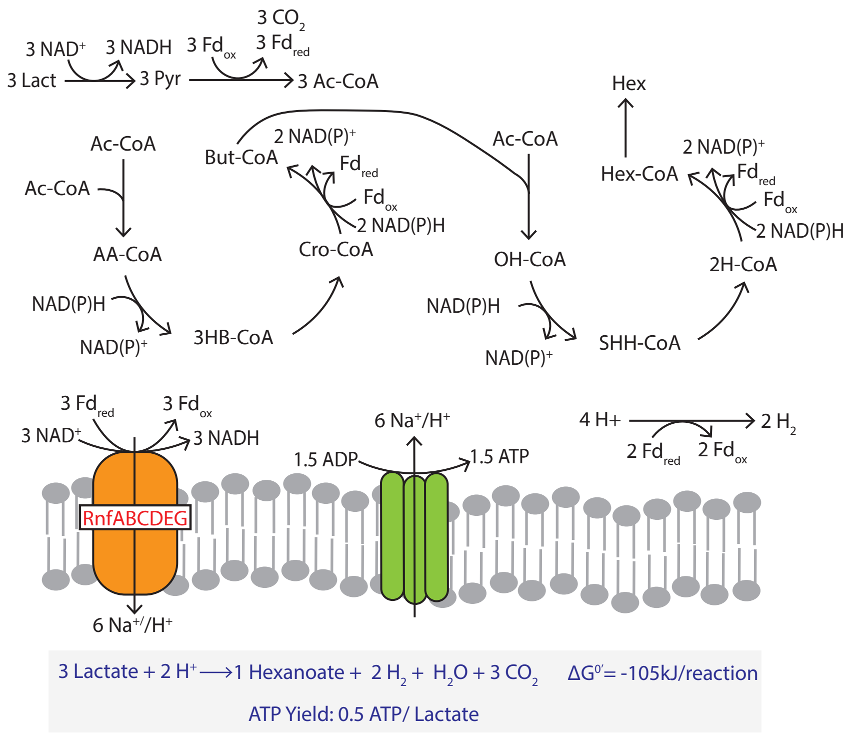

Fiq S7.24 (Eq. 24)

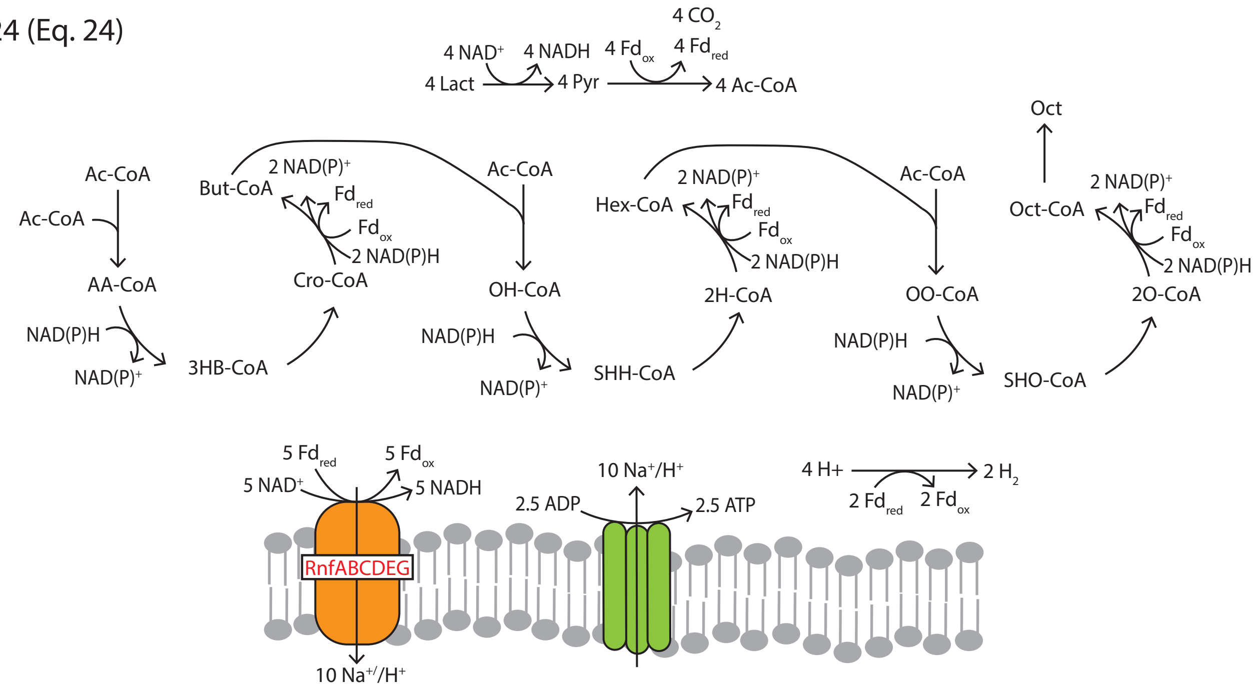

4 Lactate $+ 3 H^+ \longrightarrow 1$ Octanoate $+ 2 H_2 + 2 H_2O + 4 CO_2$ $\Delta G^{0'} = -156$ kJ/reaction

ATP Yield: 0.63 ATP/ Lactate

Fiq S7.25 (Eq. 25)

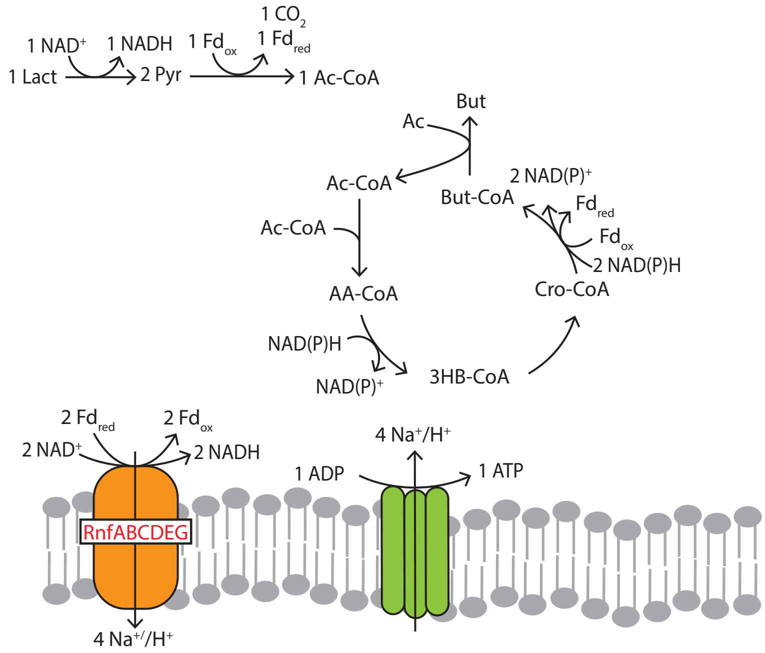

1 Lactate + 1 Acetate +1 $H^+ \longrightarrow$ 1 Butyrate + 1 CO_2 + 1 H_2O $\Delta G^{0'} = -51$ kJ/reaction

ATP Yield: 1 ATP/ Lactate

Fiq S7.26 (Eq. 26)

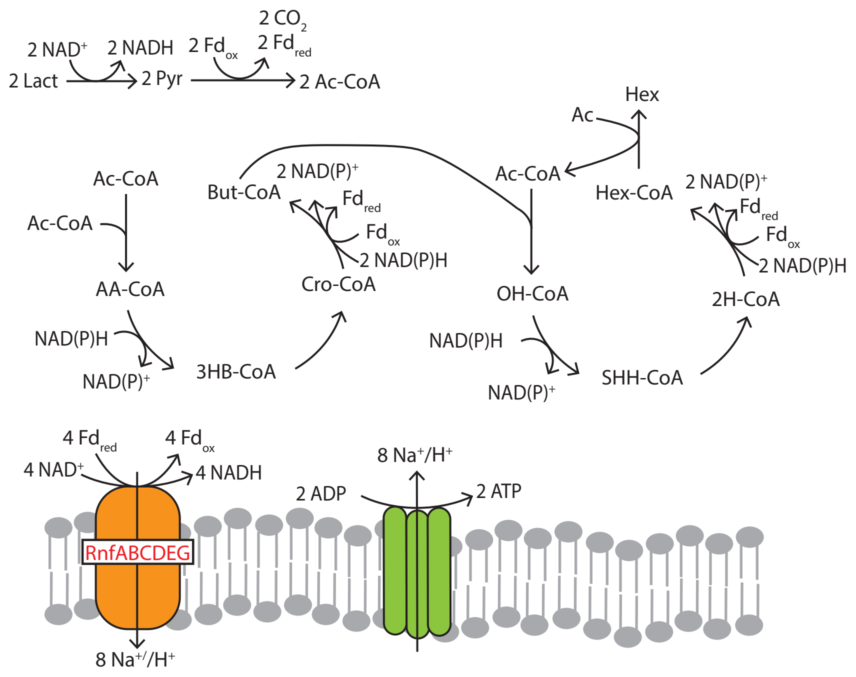

2 Lactate + 1 Acetate +2 H$^+$ ⟶ 1 Hexanoate + 2 CO_2+ 2 H_2O $\Delta G^{0'}$= -102 kJ/reaction

ATP Yield: 1 ATP/ Lactate

Fiq S8.27 (Eq. 27)

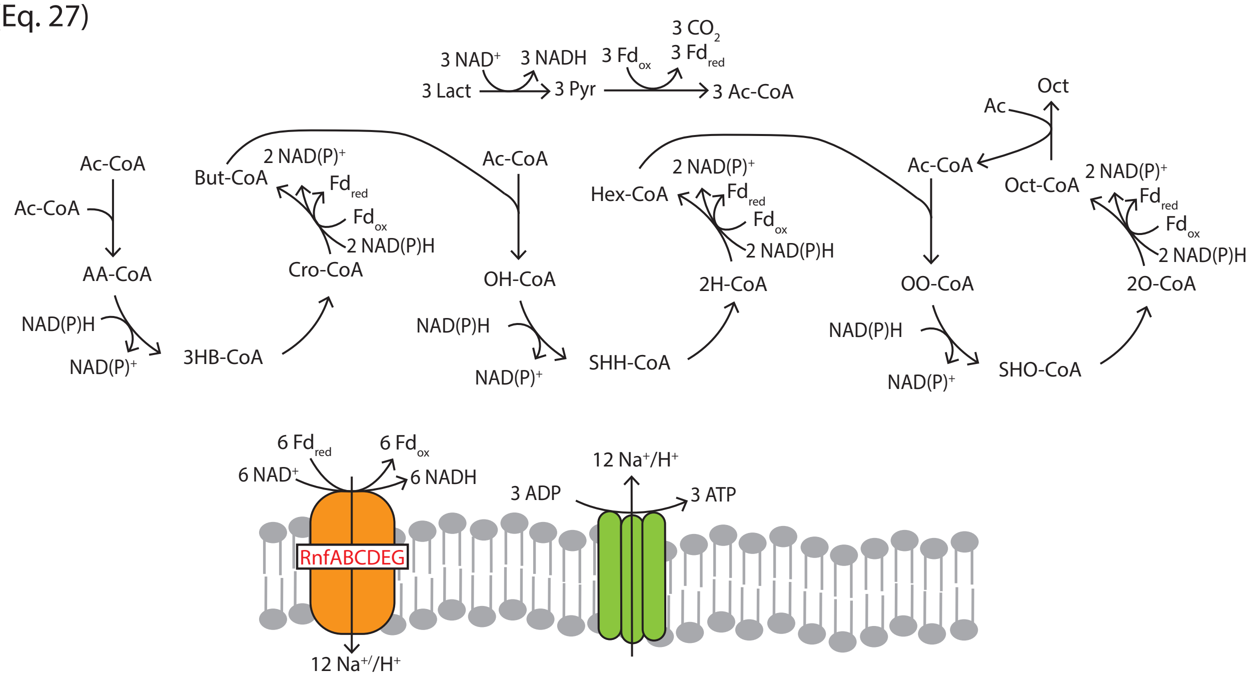

3 Lactate + 1 Acetate +3 H$^+$ ⟶ 1 Octanoate + 3 CO_2+ 3 H_2O $\Delta G^{0'}$= -156 kJ/reaction

ATP Yield: 1 ATP/ Lactate

Fiq S7.28 (Eq. 28)

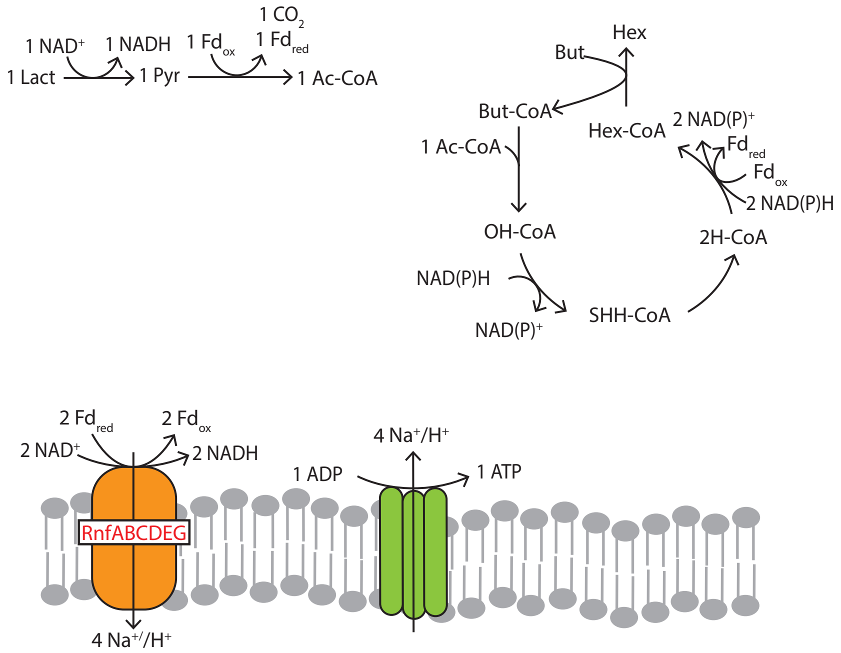

1 Lactate + 1 Butyrate +1 H^+ ⟶ 1 Hexanoate + 1 CO_2 + 1 H_2O $\Delta G^{0'}$ = -51 kJ/reaction

ATP Yield: 1 ATP/ Lactate

Fiq S7.29 (Eq. 29)

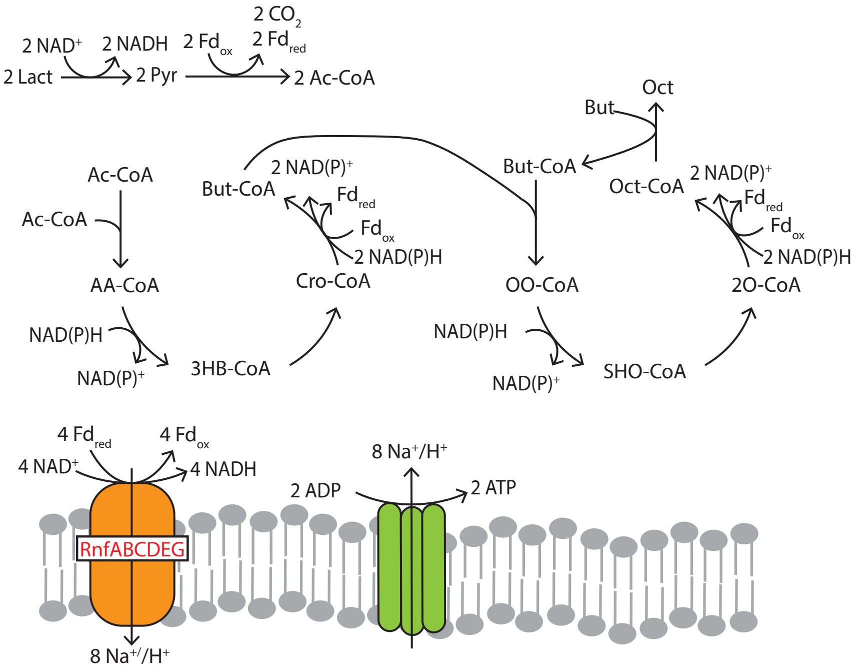

2 Lactate + 1 Butyrate +2 H^+ ⟶ 1 Octanoate + 2 CO_2 + 2 H_2O

ATP Yield: 1 ATP/ Lactate $\Delta G^{0'}$ = -103 kJ/reaction

Fiq S7.30 (Eq. 30)

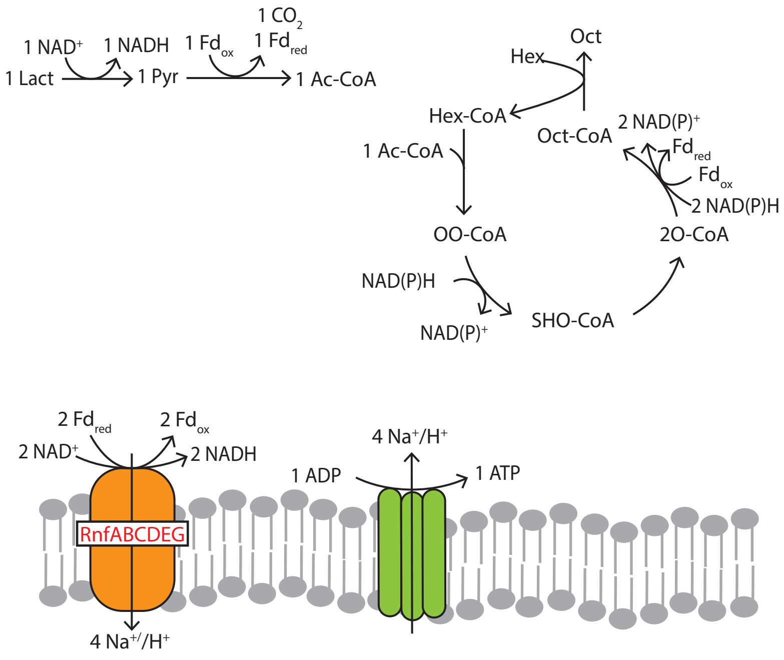

1 Lactate + 1 Hexanoate +1 H^+ ⟶ 1 Octanoate + 1 CO_2 + 1 H_2O

$\Delta G^{0\prime}$= -54 kJ/reaction

ATP Yield: 1 ATP/ Lactate

Fiq S7.31 (Eq. 31)

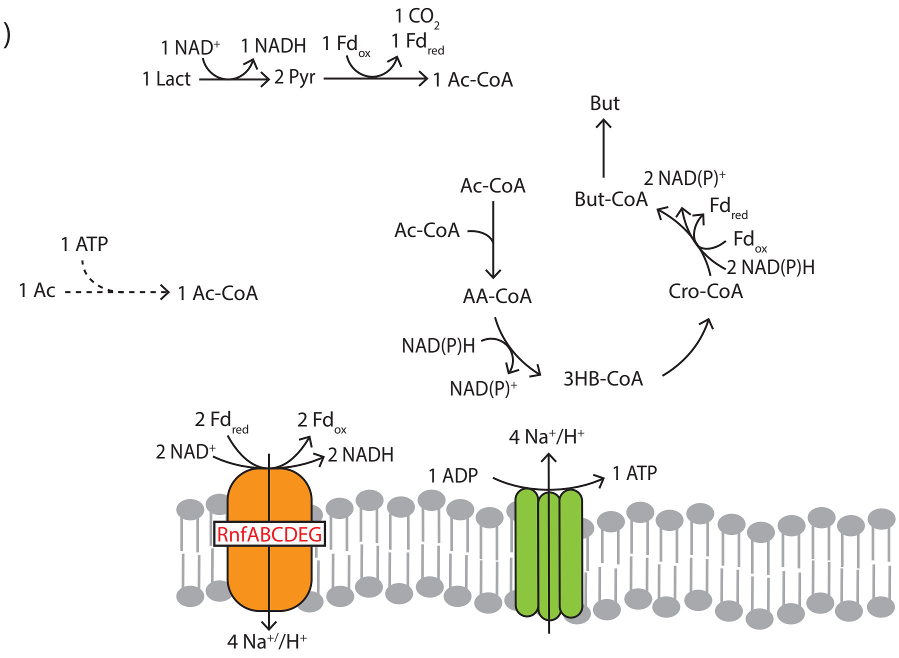

1 Lactate + 1 Acetate +1 H^+ ⟶ 1 Butyrate + 1 CO_2 + 1 H_2O

$\Delta G^{0\prime}$= -51 kJ/reaction

ATP Yield: 0 ATP/ Lactate

Fiq S7.32 (Eq. 32)

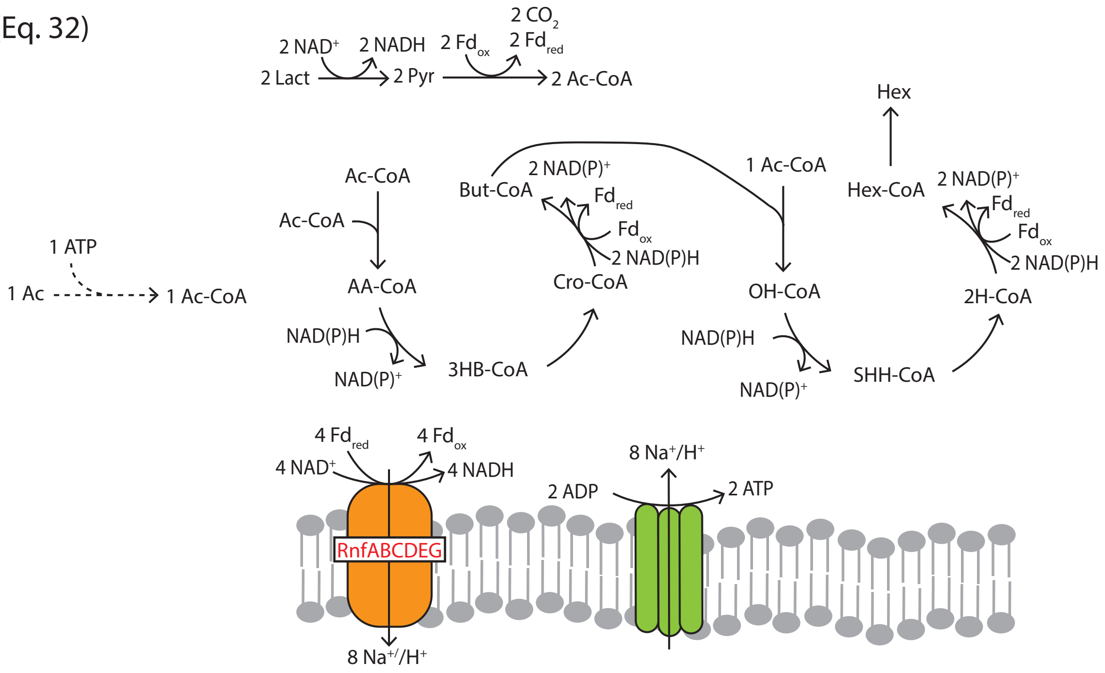

2 Lactate + 1 Acetate +2 H^+ ⟶ 1 Hexanoate + 2 CO_2 + 2 H_2O $\Delta G^{0'}$= -102 kJ/reaction

ATP Yield: 0.5 ATP/ Lactate

Fiq S7.33 (Eq. 33)

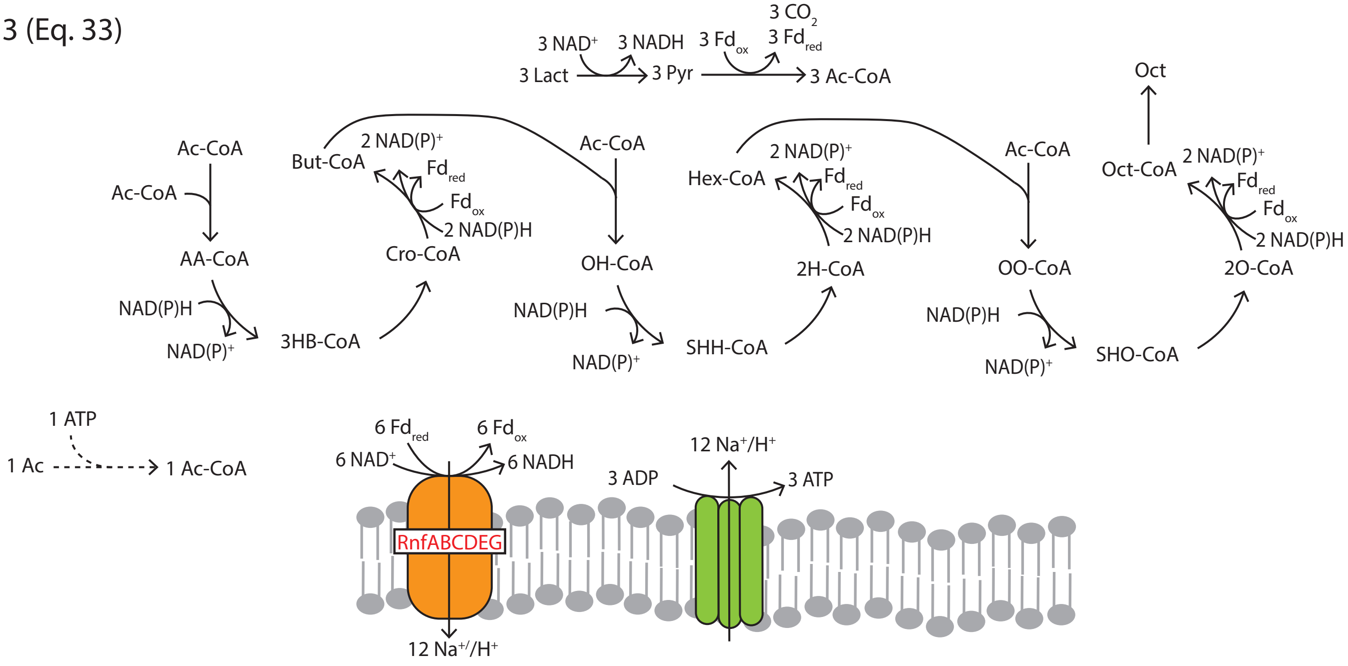

3 Lactate + 1 Acetate +3 H^+ ⟶ 1 Octanoate + 3 CO_2 + 3 H_2O $\Delta G^{0'}$= -156 kJ/reaction

ATP Yield: 0.67 ATP/ Lactate

Fiq S7.34 (Eq. 34)

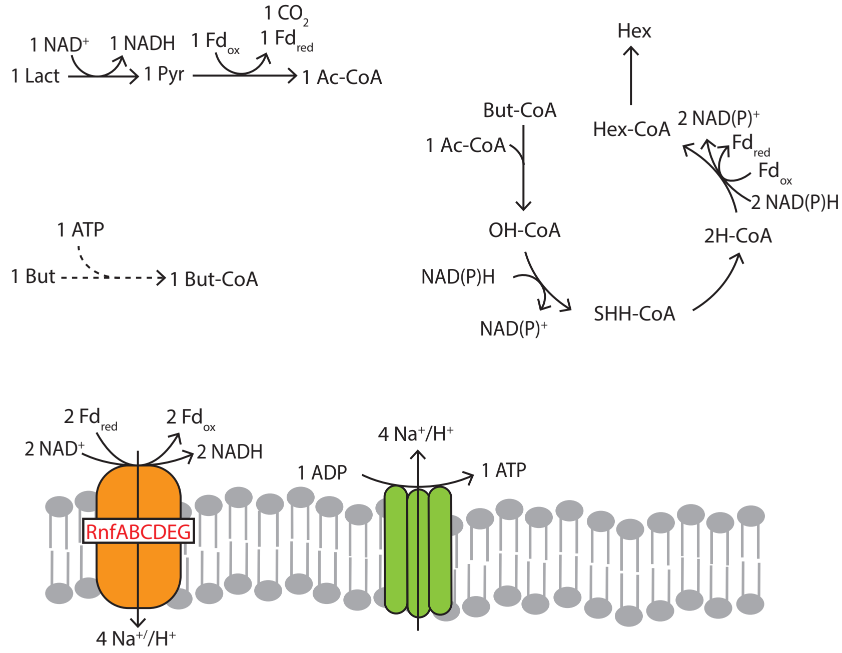

1 Lactate + 1 Butyrate +1 H^+ ⟶ 1 Hexanoate + 1 CO_2 + 1 H_2O $\Delta G^{0'}$ = -51 kJ/reaction

ATP Yield: 0 ATP/ Lactate

Fiq S7.35 (Eq. 35)

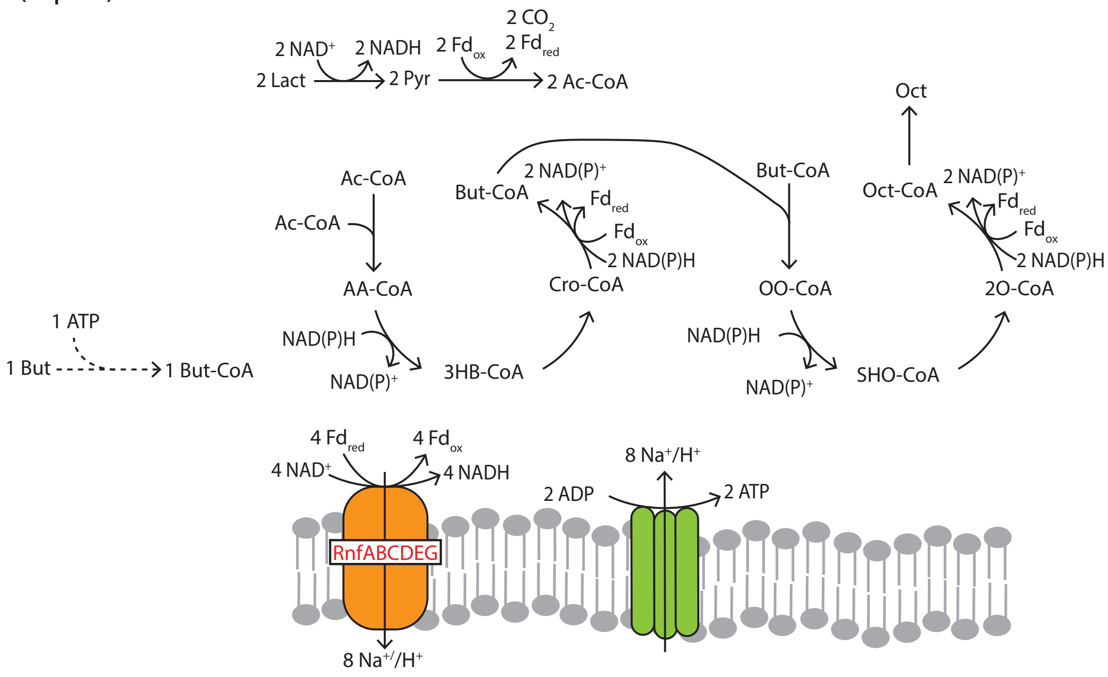

2 Lactate + 1 Butyrate +2 H^+ ⟶ 1 Octanoate + 2 CO_2 + 2 H_2O

ATP Yield: 0.5 ATP/ Lactate $\Delta G^{0'}$ = -103 kJ/reaction

Fiq S7.36 (Eq. 36)

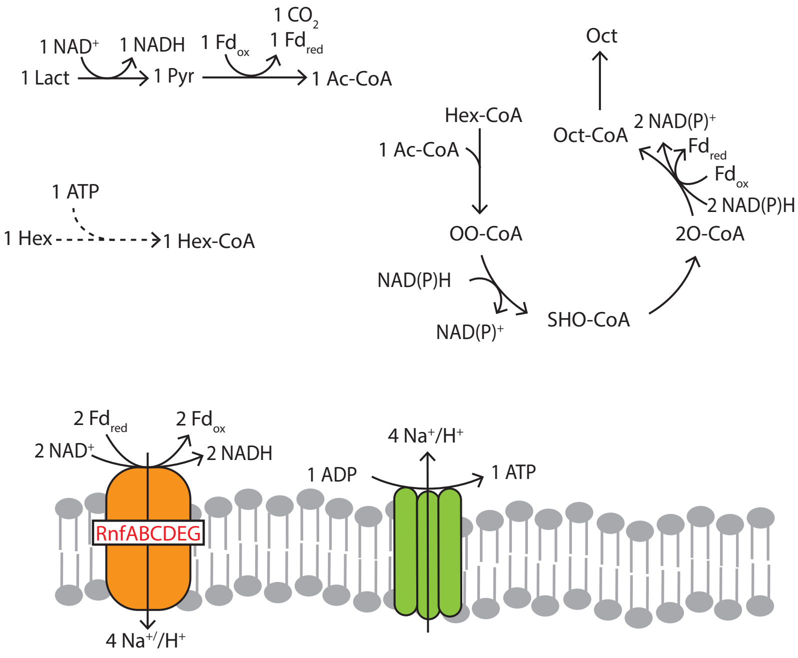

1 Lactate + 1 Hexanoate +1 H^+ $\longrightarrow$ 1 Octanoate + 1 CO_2+ 1 H_2O

$\Delta G^{0'}$= -54 kJ/reaction

ATP Yield: 0 ATP/ Lactate
