# Supplementary material for: Metatranscriptomic and Thermodynamic Insights into Medium-Chain Fatty Acid Production Using an Anaerobic Microbiome
Source: mSystems. 2018 Nov 20;3(6):e00221-18. doi: 10.1128/mSystems.00221-18 (PMC6247018; doi:10.1128/mSystems.00221-18)
Supplement: TABLE S1 [file sys006182291st1.docx]

| **Bin ID** | **Taxonomy** | **Completeness (%)** | **Contamination (%)** | **Genome size (bp)** | **# Scaffolds** | **N50** | **GC (%)** | **Predicted Genes** |
| --- | --- | --- | --- | --- | --- | --- | --- | --- |
| LCO1 | Firmicutes; Clostridia; Clostridiales; Lachnospiraceae; Shuttleworthia | 95.4 | 0.0 | 2,106,912 | 44 | 103,964 | 45.7 | 1,900 |
| EUB1 | Firmicutes; Clostridia; Clostridiales; Eubacteriaceae; Pseudoramibacter | 97.8 | 0.2 | 2,002,609 | 35 | 142,846 | 51.2 | 1,857 |
| COR1 | Actinobacteria; Actinobacteria; Coriobacteriales; Coriobacteriaceae; Olsenella | 99.2 | 0.8 | 2,512,349 | 225 | 22,880 | 59.0 | 2,358 |
| COR2 | Actinobacteria; Actinobacteria; Coriobacteriales; Coriobacteriaceae; Olsenella | 100 | 1.6 | 2,422,853 | 155 | 34,678 | 64.8 | 2,185 |
| COR3 | Actinobacteria; Actinobacteria; Coriobacteriales; Coriobacteriaceae; Olsenella | 98.4 | 7.4 | 3,647,413 | 533 | 13,368 | 55.5 | 4,068 |
| LAC1 | Firmicutes; Bacilli; Lactobacillales; Lactobacillaceae; Lactobacillus | 99.5 | 1.1 | 2,633,889 | 18 | 640,122 | 43.6 | 2,567 |
| LAC2 | Firmicutes; Bacilli; Lactobacillales; Lactobacillaceae; Lactobacillus | 99.4 | 1.6 | 3,179,174 | 79 | 122,889 | 40.5 | 2,989 |
| LAC3 | Firmicutes; Bacilli; Lactobacillales; Lactobacillaceae; Lactobacillus | 99.2 | 1.4 | 2,704,063 | 174 | 29,509 | 43.0 | 2,731 |
| LAC4 | Firmicutes; Bacilli; Lactobacillales; Lactobacillaceae; Lactobacillus | 98.9 | 1.3 | 3,335,227 | 95 | 86,779 | 40.2 | 3,150 |
| LAC5 | Firmicutes; Bacilli; Lactobacillales; Lactobacillaceae; Lactobacillus | 80.1 | 0.8 | 1,487,044 | 181 | 12,363 | 46.1 | 1,524 |
